# The editable landscape of the yeast genome reveals hotspots of structural variant formation

**DOI:** 10.1126/sciadv.ady9875

**Published:** 2025-10-31

**Authors:** Shengdi Li, Sibylle C. Vonesch, Kevin R. Roy, Chelsea Szu Tu, Friederike Steudle, Michelle Nguyen, Cosimo Jann, Lars M. Steinmetz

**Affiliations:** ^1^ Genome Biology Unit, European Molecular Biology Laboratory (EMBL), Heidelberg 69117, Germany.; ^2^ Stanford Genome Technology Center, Stanford University, Palo Alto, CA 94304, USA.; ^3^ Department of Genetics, Stanford University School of Medicine, Stanford, CA 94305, USA.

## Abstract

It is unclear how CRISPR editing outcomes vary across the genome and whether undesirable events such as structural variants (SVs) are predictable or preventable. To define a genome-wide map of editability, we performed whole-genome sequencing on 1875 budding yeast clones edited across 16 chromosomes by CRISPR-Cas9 and donor-templated repair. We found that unintended edits, including short indels and SVs, were enriched in specific genomic and sequence contexts. We developed a predictive model, SCORE (System for CRISPR Outcome and Risk Evaluation), which revealed 4.8% of the genome as SV prone, consisting of 562 SV hotspots. Donor repair-enhancing strategies suppressed SV formation in regions with moderate, but not high, predicted risk. Applying SCORE to the Sc2.0 synthetic yeast genome revealed a markedly altered SV landscape due to the removal of endogenous repetitive elements and the insertion of loxP sites. Our study provides the genome-scale map of SV hotspots after CRISPR editing and predictive and experimental tools to mitigate their formation.

## Teaser

Some parts of the genome are intrinsically difficult to edit, necessitating systematic approaches to elucidate their patterns.

## INTRODUCTION

CRISPR RNA-guided programmable nucleases have revolutionized research, medicine, and biotechnology by enabling precise and efficient editing of the genome. However, the potential for unintended mutations at on- and off-target sites continues to raise concerns for basic research (*1*), therapeutic (*2*), and industrial applications (*3*). CRISPR editing systems trigger genome editing by inducing either single-strand breaks (SSBs) or double-strand breaks (DSBs). DSBs can lead to disruption of target genes through small insertions or deletions via nonhomologous end joining (NHEJ) or microhomology-mediated end joining (MMEJ) (*4*). DSBs can also be harnessed for incorporation of designed sequence changes by homology-directed repair (HDR) with an exogenous template. However, in some instances, CRISPR-induced DSBs can lead to unintended structural variants (SVs), including large deletions, translocations, loss of chromosome arms, and partial aneuploidies (*5*, *6*). These SVs can have serious adverse effects as they can lead to gene loss, silencing, or altering of gene function, and can precipitate chromosomal instability and chromothripsis (*7*). Even nickase-based systems, including base and prime editors, induce transient DSB formation at nicked sites that can yield SVs at both on-target and off-target sites (*8*–*11*). Understanding why SVs occur and predicting regions at risk for SV formation is thus relevant for all genome editing approaches. While existing tools have enabled predictions of certain editing outcomes, such as editing efficiency (*12*, *13*) and the spectrum of small indels resulting from NHEJ (*14*) and MMEJ (*15*), no tool is presently capable of predicting large-scale chromosomal rearrangements or large deletions.

Although there are isolated reports of SVs occurring with a variety of CRISPR systems, comprehensive, genome-wide surveys characterizing their frequency and genomic distribution are currently missing. This gap largely stems from the inherent limitations of conventional genotyping methods [e.g., polymerase chain reaction (PCR)–based target sequencing] in detecting SVs and a lack of approaches capable of characterizing unexpected editing outcomes such as SVs across the genome in an unbiased fashion. Consequently, it is unclear how frequently SVs arise across any genome in response to CRISPR editing, whether certain regions are at a particularly high risk, and if such loci can be predicted based on sequence and chromosomal context. In this study, we tackle this question using the budding yeast model system, as its relatively modest genome size allows for comprehensive whole-genome sequence (WGS) assessment across many genomic sites. We analyzed WGS outcomes for thousands of edits spanning all 16 nuclear chromosomes and found that most sites are edited correctly and that off-target indels by Cas9 are virtually nonexistent. Unexpectedly, however, we find that a subset of target sites is highly prone to forming SVs. We show that these SVs arise from competing local and/or distal sequence repetitiveness and built a machine learning model termed SCORE (System for CRISPR Outcome and Risk Evaluation) to map the locations of all SV hotspots in the genome as well as to assess the likelihood for correct editing at all SpCas9 target sites.

## RESULTS

### Mapping intended and unintended CRISPR-Cas9 editing outcomes genome-wide

**Fig. 1.**
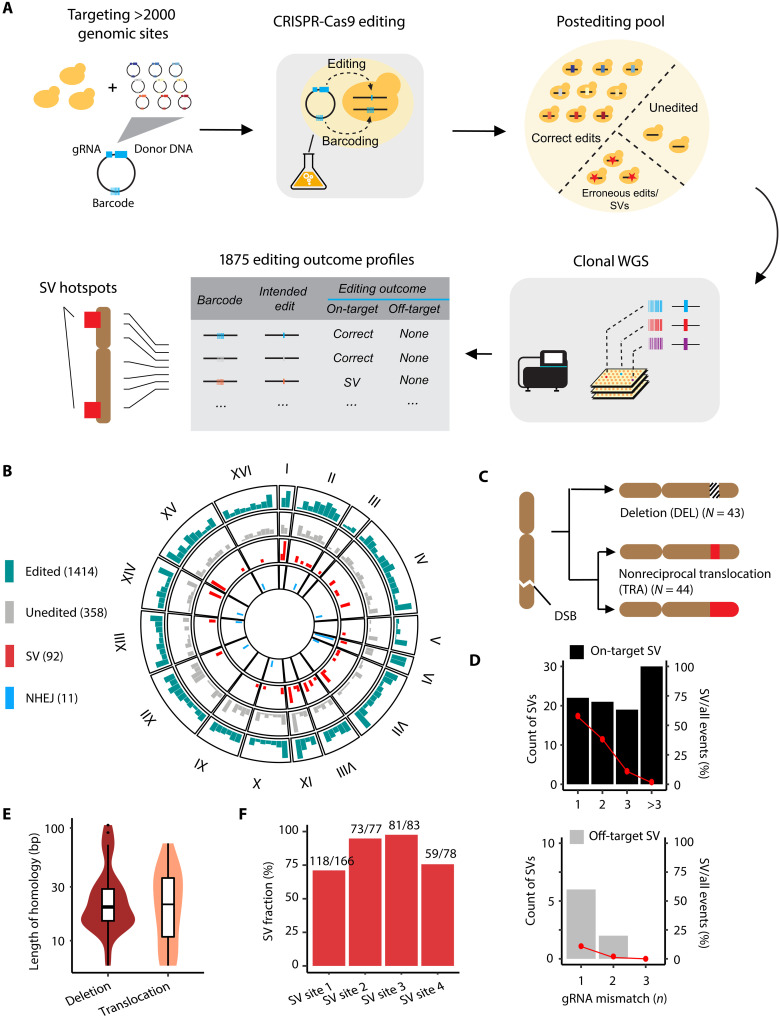
Characterization of donor-templated CRISPR-Cas9 editing outcomes throughout the yeast genome. (**A**) Workflow for mapping and characterizing CRISPR-Cas9 editing events. (**B**) Genomic distribution of 1875 characterized on-target events. Height of bars indicates frequency of editing events detected within 100-kb bins of the genome. The frequency range was normalized to display values between 0 and the maximum value for each editing outcome category. (**C**) Subtypes of SVs observed following Cas9-mediated double-strand breaks. (**D**) On-target (top panel) and off-target SVs (bottom panel) stratified by gRNA mismatch. For the on-target panel, the mismatch number corresponds to that of the off-target site with the lowest number of mismatches. Bars represent absolute count of events (left vertical axis), while the dotted lines indicate percentage of SV among all detected events for each mismatch number (right vertical axis). (**E**) Distribution of homology length (bp) without mismatches present at SV breakpoints. (**F**) Reproducibility of SV formation investigated for four SV-causing guide donor pairs. Number above the bars indicates the absolute counts of SV-containing clones over all sequenced clones.

To explore the spectrum of CRISPR-Cas9 editing outcomes genome-wide, we used a multiplexed HDR template–based CRISPR editing approach we previously developed termed MAGESTIC (*16*) to edit a laboratory strain of *Saccharomyces cerevisiae* at sites spread across the genome (Fig. 1A). To target the entire genome in an unbiased fashion, we designed a library of thousands of single-nucleotide and multinucleotide variants (SNVs/MNVs) and short indels naturally present in the species and which are spread throughout all 16 chromosomes. First, we constructed a pool of paired guide RNA (gRNA) and donor DNA (guide-donor) plasmids tagged with short, random DNA barcodes (Fig. 1A). The barcodes serve as unique edit identifiers and are integrated at a dedicated locus in the genome after editing. We used robotic colony picking and recombinase-directed indexing (REDI) (*17*) to pick 2260 haploid clones with barcodes corresponding to verified guide-donor sequences for whole-genome sequencing (WGS). The picked clones encompassed three successive versions of the MAGESTIC system (v1.1, v1.2, and v1.3), each incorporating iterative improvements in editing efficiency (Materials and Methods). Next, we prepared WGS libraries with a low-cost and efficient method we previously developed that bypasses the need for genomic DNA extraction (*18*). We sequenced each genome at 5× coverage and mapped the intended and unintended mutations in each individual genome with a computational pipeline geared toward assessing intended edits, short indels, and various types of SVs (fig. S1A and Materials and Methods).

A total of 1875 clones harbored sufficient coverage at the target regions for calling editing outcomes (Fig. 1B and table S1). A total of 1414 clones exhibited HDR template–encoded variants consistent with the intended edits predicted by the genome-integrated barcodes. Of 346 MNV-intended edits, 57 (16.5%) did not incorporate all the SNVs present in the donor (fig. S1B), with variant incorporation rate dropping with distance from the “NGG” protospacer adjacent motif (PAM) (fig. S2A). We also noticed a subset of clones (139 of 1414) that harbored not only the designed edit but also distal SNVs. These variants were present in the cloning stage of the guide-donor plasmid library and likely arose during oligonucleotide synthesis (fig. S2B and Supplementary Text). The likelihood of incorporation of a PAM-distal variant or synthesis error decreased as a function of distance from adjacent variants, reaching 50% incorporation rate at around 10 bp distance and falling below 10% after 20 bp (fig. S2C).

Overall, 358 of 1875 clones carried the wild-type sequence at their target sites. Notably, these unedited clones were overrepresented in earlier versions of the MAGESTIC system (fig. S3), with the most recent version (MAGESTIC v1.3) exhibiting an 82.7% correct edit rate (1094 of 1323 sites). NHEJ indels were present at low levels across all systems (11 of 1875 clones, or 0.59%), consistent with the NHEJ pathway being less error prone in *S. cerevisiae* relative to other organisms (*19*) and with few clones surviving via NHEJ indel formation (*20*). We observed no evidence of gRNA-dependent off-target indels or point mutations (fig. S4). gRNA-independent sequence changes were present at a level consistent with the expected background rate of mutations arising from propagation in cell culture (*21*) (fig. S4). Collectively, our results demonstrate that unintended off-target indels and SNVs after CRISPR nuclease-based genome editing are exceedingly rare in *S. cerevisiae* and that editing is highly efficient and precise across the majority of sites.

### SVs are induced at target sites

In addition to the wild-type sequence and HDR- and NHEJ-mediated edits, numerous clones exhibited substantial loss of sequencing coverage at the target sites relative to the rest of the genome (figs. S1A and S5A). The specific depletion of mapped reads at target sites was suggestive of large-scale genomic changes such as SVs. To better understand the occurrence of these events, we developed a pipeline to systematically detect SVs across 2260 targeted regions and 654 computationally predicted off-target regions (with 1, 2, or 3 mismatches to the corresponding gRNA) (Materials and Methods). Our analysis identified a total of 92 target sites with SVs (Fig. 1B), comprising 44 large deletions (DELs), 43 nonreciprocal translocations (TRAs), and 5 unclassified SVs. TRAs could be further subdivided on the basis of whether replacement of the target sequence reached the telomere (Fig. 1C and fig. S5A). Complete chromosomal arm replacement events were detected exclusively in (sub)telomeric regions, whereas incomplete replacements were found throughout chromosomes (fig. S6A). Both events led to loss of sequence at the targeted site and duplication of sequence from another chromosomal location (fig. S6B and table S2). As SV detection is influenced by sequencing depth, samples with lower coverage (<5×) likely underestimated SV fractions. The true SV rate in the editing pool is therefore expected to be slightly higher than the 4.9% (92 of 1875) estimated from all strains, approaching 7% in samples with >10× coverage (24 of 345) (fig. S7). In addition to these 92 clones, we observed 8 clones with SVs at predicted off-target sites with one or two mismatches to the gRNA, yielding a total of 100 SVs across the entire dataset (Fig. 1D and table S3). Notably, six of eight off-target SVs occurred in regions with a single mismatch to the gRNA, two of eight with two mismatches, and none with three mismatches, underlining the risk of off-target cleavage at sites with high sequence similarity to the target.

More than half of the DEL and TRA breakpoints, defined by the mismatched base at the end of the modified sequence within the target region (fig. S5B), are flanked by ≥20 bp of perfect homology between the target and a distal genomic region (Fig. 1E). This suggests that SV formation is not a pervasive outcome of DNA repair but rather is enriched in genomic regions where endogenous homologous sequences compete with the exogenous donor HDR template. Further inspection of the target loci yielding SVs revealed local repetitive sequences present as tandem repeats in the case of deletions and distal repetitive sequences in the case of nonreciprocal translocations. As confirmed by recloning, editing, and sequencing of four target sites yielding SV clones (Supplementary Text and fig. S6C), the SV rate of a single guide-donor pair ranged from 71.1 to 97.6% (Fig. 1F and table S4), substantially exceeding the genome-wide rate of 4.9% (92 of 1875 clones). Furthermore, sites with SVs exhibited slightly greater chromatin accessibility (versus unedited sites, Wilcoxon’s rank sum test’s *P* = 0.029, *N* = 178) (fig. S6D) and higher H2A S129 phosphorylation (H2AS129ph) level (versus unedited sites, Wilcoxon’s rank sum test’s *P* = 2.7 × 10^−5^, *N* = 178) (fig. S8). H2AS129ph (functionally equivalent to human H2AS139ph, also known as γH2AX) is transiently induced after DNA damage (*22*) and found at higher levels in the S-G_2_ phases specifically in regions prone to DNA replication stress such as the subtelomeric regions (*23*). We reasoned that the correlation of SV incidence with H2AS129ph is likely a consequence of the sequence repetitiveness in these regions, which leads to a high recombination rate and genetic instability (*24*). Together, these findings indicate that Cas9-induced SVs are context dependent and driven primarily by HDR in the yeast system. Their tendency to arise predominantly in repetitive regions suggests that these occurrences may, in fact, be predictable. This contrasts with the limited studies on SV prevalence in mouse and human cells, where SV formation shows a predisposition to MMEJ pathways and where outcomes are highly heterogenous and do not appear to follow any predictable pattern based on the locations or lengths of microhomology sequences (*25*, *26*).

### Development of a machine learning model for predicting Cas9-induced SVs genome-wide

**Fig. 2.**
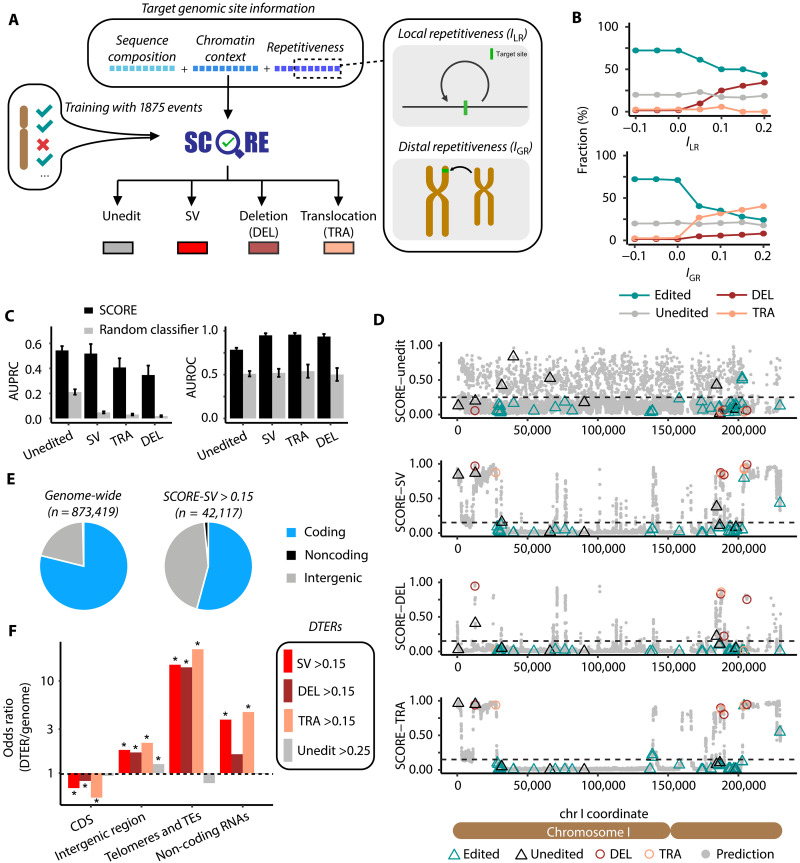
SCORE predicts genomic regions prone to unintended editing consequences. (**A**) Schematic representation of the SCORE model input and output. (**B**) Distribution of editing outcomes in repetitive genomic regions. Repetitiveness of the targeted regions is measured by *I*_LR_ (top panel) and *I*_GR_ (bottom panel). Dotted lines denote the trend in outcome composition shift as the threshold of sequence repetitiveness increases. (**C**) Performance of SCORE models evaluated by repeated *k*-fold cross-validation, compared to a random classifier. Error bars indicate standard deviations of the AUPRC/AUROC values across 15 cross-validation datasets (5 repeats × 3 folds). (**D**) Predicted SCOREs for unintended outcomes across 15,291 NGG PAM sites on S288C chromosome I. Sites with experimentally confirmed outcomes (edited, unedited, DEL: deletion, and TRA: translocation) were highlighted. (**E**) Composition of SV-prone genomic sites (SCORE-SV > 0.15) compared to the entire genome. (**F**) Four types of DTERs and their composition of genomic regions. Chi-square tests were performed to indicate enrichment or depletion of each region type in DTER relative to genomic background, followed by Bonferroni correction for the resulting *P* values. *P value <10^−4^. DTER, difficult-to-edit region; TE, transposable elements.

We developed SCORE (https://apps.embl.de/score/) to predict the likelihood of an SV or lack of editing for every SpCas9 target site in the *S. cerevisiae* genome. SCORE is an ensemble of gradient-boosted decision tree (GBDT) models trained on the 1875 characterized events in our clones (Fig. 2A). Four classifiers were trained independently to distinguish correct edits from unedited sites, DELs, or TRAs, incorporating relevant context information encompassing target sequence composition, chromatin status and sequence repetitiveness (tables S5 and S6 and Supplementary Text).

Given the observation that endogenous sequence homology appeared to be driving DELs and TRAs (Fig. 1E), we incorporated two different metrics into SCORE to distinguish between local and distal repetitive sequences (Fig. 2A): the index of local repetitiveness (*I*_LR_ or *I*_r_) (*27*) and the index of global repetitiveness (*I*_GR_), respectively (Materials and Methods). As expected, *I*_LR_ and *I*_GR_ computed from a 500-bp window surrounding the target site correlated with SV rates, with DEL outcomes increasing with *I*_LR_ and TRA outcomes with *I*_GR_ (Fig. 2B). To account for both short- and long-range sequence context, we additionally encoded the *I*_LR_ and *I*_GR_ using gradients of window sizes ranging from 50 bp to 10 kb to encompass the SV sizes observed in our dataset (up to ~3 kb deletion) (fig. S9).

The model architecture and parameters of SCORE were determined by benchmarking the model using repeated (*n* = 5) threefold cross-validation over the training data (Materials and Methods). We compared four machine learning algorithms (random forest, GBDT, and L1- and L2-regularized logistic regressions) and found GBDT to be superior (fig. S10A). To mitigate the class imbalance of SVs and unedited clones compared to correctly edited clones, the correct edits were downsampled accordingly for the training data (fig. S10, B and C). Following model tunings and optimizations, the finalized SV predictor achieved an AUROC (Area Under the Receiver Operating Characteristic Curve) of 0.949 and an AUPRC (Area Under the Precision-Recall Curve) of 0.518 (Fig. 2C). All four SCORE submodels substantially outperformed random classifiers (Fig. 2C). Analysis of feature importance revealed long T homopolymers (*16*, *28*) and gRNA sequence composition as the top variables predicting the absence of editing (fig. S11). The editing system influenced baseline editing efficiency and acted as a confounding factor to the SCORE-unedit model (fig. S3A). Local and distal sequence repetitiveness were the top variables predicting SVs, with no detectable impact from the editing system (fig. S11). This finding aligns with the observation that SV rates remained relatively consistent across the three editing systems of varying efficiency (fig. S3A), suggesting that SV formation becomes the dominant repair outcome in repetitive regions, obfuscating the editing machinery enhancements optimized for nonrepetitive regions.

### A map of the editable regions in the *S. cerevisiae* reference genome

Using SCORE, we conducted a genome-wide survey of editable regions encompassing all 873,419 NGG motifs with unique spacers on 16 yeast chromosomes, excluding 66,538 sequences with perfect off-target matches (gRNAs that can bind to >1 genomic target without mismatches). The predicted scores for the absence of editing were scattered along the chromosomal axis, suggesting that local sequence context plays a major role over larger regional effects (Fig. 2D). In contrast, sites with high SV probabilities were clustered into a handful of hotspots per chromosome (Fig. 2D).

The genomic distribution of SV scores showed a skewed, right-tailed distribution, reflecting the low rate of SV formation for the majority of the genome, with only a small portion exhibiting elevated risks. This hypothesis aligns with the low fraction of SVs observed in our pooled editing experiment (3.8 to 7.4%, fig. S3A) but high SV rate at individual target sites (Fig. 1E). On the basis of the empirical distribution of each predictive score, we established cutoffs to identify outliers within the tails of each distribution (fig. S12; SV score > 0.15; DEL score > 0.15; TRA score > 0.15; unedited score > 0.25) and categorize these sites as difficult-to-edit regions (DTERs). This approach identified 4.8% of the genome susceptible to SV formation upon Cas9 cleavage (1.7% for DEL and 3.1% for TRA) and 11.1% of sites as likely to remain unedited (table S7). By grouping sites in proximity with SV scores >0.15, we identified 562 SV hotspots across 16 yeast chromosomes, with an average cluster size of 2 kb and 57.9 PAM sites per cluster (table S8). Of the regions at risk for SVs, 54.2% overlapped with protein-coding genes, indicating a depletion of SV hotspots within the 78.9% of the genome that codes for proteins (Fig. 2E). The distribution of SV scores in essential genes and stress-responsive TATA box–containing genes (*29*) (Materials and Methods) showed no trend of increase or decrease compared to all coding regions (fig. S13). It is important to note, however, that SCORE does not consider the phenotypic impact of the editing outcomes, and it is possible, if not likely, that many of the SVs predicted in essential genes may not lead to viable outcomes. As expected, SV-prone loci were more prevalent in telomeric and transposable elements, as well as tRNA genes due to their repetitive nature (Fig. 2F and fig. S13). The ribosomal RNA genes, present as a long array of 100 to 150 tandem repeats (*30*), were absent from our analysis as no gRNA sequences can uniquely target a single copy.

We further examined the relationship between our predicted SV landscape and naturally occurring SVs that have arisen during the evolution of *S. cerevisiae* genome, as defined by a collection of 142 telomere-to-telomere assemblies of yeast strains (*31*). We observed not only an overall elevation in prediction scores at natural SV sites but also a colocalization between natural SV breakpoints and local peaks in the prediction score (fig. S14). Although the SVs identified in our study were induced by artificial DNA DSBs (i.e., by the CRISPR-Cas9 system), this finding suggests that the underlying DNA repair outcome preferences are likely shared with those driving natural SV formation. Moreover, these repair outcome preferences, as observed in our laboratory strain, appear to be broadly conserved across natural *S. cerevisiae* populations and may have played a role in shaping the distribution of SVs throughout yeast genome evolution.

### Boosting HDR efficiency reduces SV formation in DTERs

**Fig. 3.**
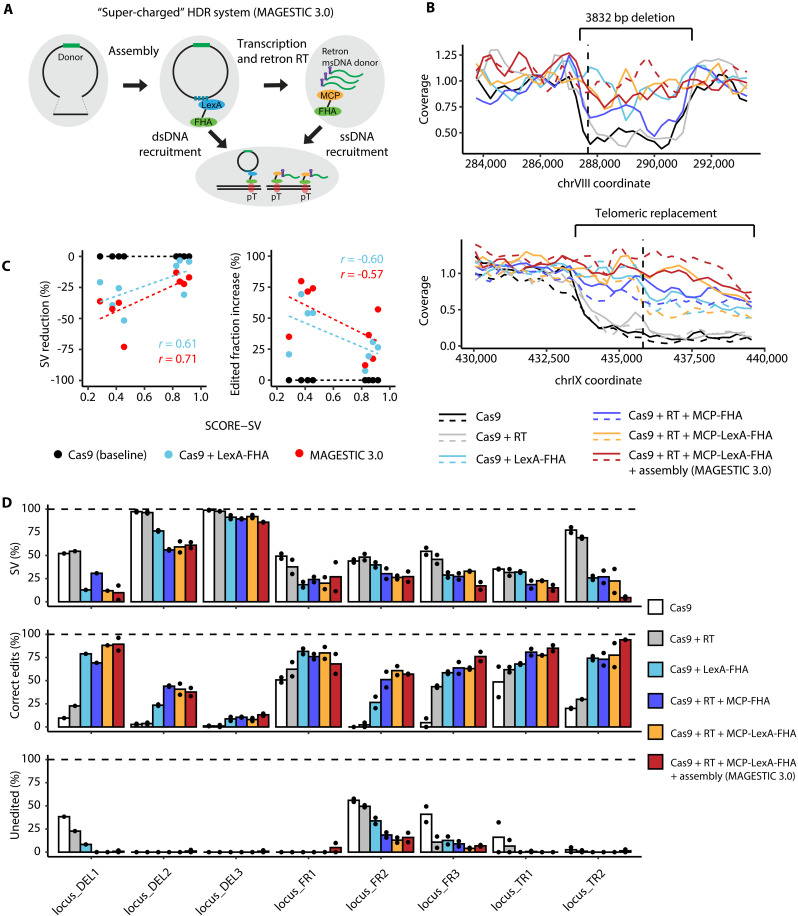
HDR enhancement reduces SV formation. (**A**) Scheme of HDR enhancement techniques used in the MAGESTIC 3.0 system. (**B**) The reduction in mapping coverage at each target (top: DEL1, bottom: TR2) was used to determine the fraction of cells with SVs. Solid and dashed lines represent two replicates of the editing experiment (*N* = 2; or *N* = 1 if only the solid line is shown). A vertical dashed line denotes the position of PAM site. Cas9, the minimal Cas9 + plasmid donor only system; RT, reverse transcriptase, for creation of ssDNA donor; FHA-LexA, FHA fused with DNA binding domain LexA, for recruitment of dsDNA donor to DSB; MCP-FHA, FHA fused with RNA binding domain MS2 for recruitment of ssDNA donor to DSB; MCP-FHA-lexA, FHA fused with both DNA and RNA binding domains, for recruitment of ssDNA and dsDNA donor to DSB; assembly, in vivo plasmid assembly. (**C**) Reduction of SV fraction (left) and increase of correctly edited fraction (right) of targets with diverse predicted SCORE-SV. *r* denotes Pearson’s correlation coefficient. (**D**) Estimated fraction of SVs (top), correctly edited (middle) and unedited alleles (bottom) for eight loci across six different editing systems.

Our results suggest that endogenous homologous sequences compete for HDR at the target site with the donor template. Therefore, we sought to assess whether these alternative repair outcomes can be reduced or prevented through specifically enhancing donor template HDR efficiency. Our dataset used an editing technique (MAGESTIC v1.3) that improves HDR efficiency by active recruitment of double-stranded DNA (dsDNA) donor templates to DSBs (*16*). This donor recruitment system is based on a fusion protein consisting of the Fkh1 forkhead-associated (FHA) domain, which recognizes phosphothreonine residues accumulating around DSBs (*32*), and the LexA DNA-binding domain (*16*) of the bacterial transcriptional repressor LexA. In a recent improvement to MAGESTIC, we “supercharged” HDR activity through combining three HDR enhancing techniques: in vivo single-stranded DNA (ssDNA) synthesis with a bacterial retron system (*33*), recruitment of plasmid dsDNA and retron-generated ssDNA templates to DSBs via an MS2 coat protein (MCP)-LexA-FHA fusion protein, and in vivo assembly of linearized donor plasmid (*34*) [Fig. 3A; MAGESTIC 3.0 (*35*)]. To assess whether SVs could be minimized through any or a combination of these HDR enhancement strategies, we compared SV formation at eight target locations across six editing systems (tables S9 and S10), spanning from a minimal setup using only plasmid donor and Cas9 (termed Cas9-only) to the full MAGESTIC 3.0 (retron + recruitment + assembly) combination system. We picked target sites reflecting a wide range of SV formation likelihoods as predicted by SCORE, including 3 DEL and 5 TRA sites. To facilitate evaluation of editing outcomes of many clones edited with the same guide-donor, we assessed SV formation via bulk WGS of the edited population of cells transformed with guide-donor plasmids (or plasmid fragments in the case of MAGESTIC 3.0) for each target (Materials and Methods).

The Cas9-only system, in the absence of any method to enhance HDR, resulted in the highest percentage of SV formation, as evidenced by the lowest mapping coverage around the target sites (Fig. 3B and fig. S15). In vivo synthesis of ssDNA templates through reverse transcription (RT) (*33*) did not lead to a substantial reduction in the SV fraction, except when ssDNA was recruited to the DSB by the MCP-LexA-FHA protein. This contrasts with previous studies where the retron ssDNA donor system substantially improved rate of donor HDR at several non–SV-forming sites (*33*, *35*), suggesting that FHA-mediated recruitment is critical for successful editing of SV-prone sites. Editing efficiency at most of the tested loci was superior in the four systems with FHA-mediated donor recruitment, regardless of template type (Fig. 3, C and D). We observed locus-specific differences in the SV fraction for different template types, with ssDNA retron donor recruitment being superior to dsDNA plasmid donor recruitment at some sites (DEL2, FR2, and TR1) but not others (DEL1 and TR2) (Fig. 3D). Overall, the MAGESTIC 3.0 system showed the highest fraction of correct donor-mediated edits and the greatest reduction in SV formation in six out of eight loci (DEL1, DEL3, FR2, FR3, TR1, and TR2). In two of the tested loci (DEL1 and TR2), the estimated SV fraction was reduced from more than 50% in the Cas9-only setup to nearly zero, with a near 100% efficiency for editing of the designed variant (Fig. 3D). In addition to preventing large SVs, the MAGESTIC 3.0 combination system also provided an advantage in overcoming smaller deletions (24 bp) at one locus (Fig. 3D, DEL2).

While HDR enhancement approaches used in the MAGESTIC 3.0 system generally reduced SV formation, some sites showed only a weak or no improvement (e.g., FR1 and DEL3). Sites with different degrees of SV reduction could be distinguished by their predicted SV scores: three sites with scores between 0.3 and 0.5 were effectively rescued, while four sites with scores above 0.8 were recalcitrant to HDR enhancement (Fig. 3C). Overall, the SV score was correlated with the degree of SV formation suppressible by the MAGESTIC3.0 system (Pearson’s *r* = 0.71, *P* = 0.051, *N* = 8). This observation suggests that SCORE can be used quantitatively to predict a continuous variation of editing outcome likelihoods genome-wide. In summary, we conclude that amplifying and recruiting exogenous donor templates to Cas9 cleavage sites can outcompete endogenous homologous sequences as repair templates and prevent undesirable repair outcomes at regions with moderate SV risk.

### Validation of SCORE across an additional 1122 clones

To test the performance of SCORE across additional genomic regions not seen in the training data, we validated our model on an independent panel of edits generated by the MAGESTIC 3.0 system. We isolated 1122 clones edited across 220 loci with 883 unique gRNA sequences and performed WGS at an average depth of ~20×. Of the 1122 clones, 488 were targeted by LbCas12a at sites flanking a 5′ TTTV PAM motif (table S11).

**Fig. 4.**
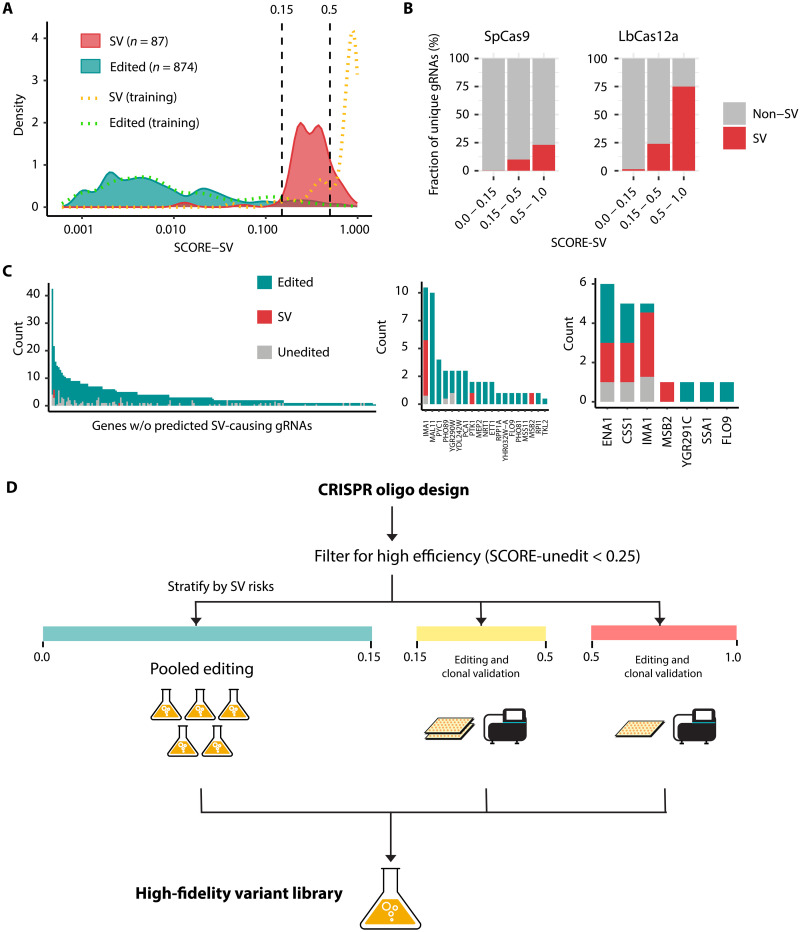
Benchmarking SCORE for CRISPR-Cas library design. (**A**) SCORE-SV distributions across 1122 clones examined by WGS. Dashed vertical lines denote thresholds of SCORE-SV at 0.15 and 0.5. (**B**) Editing outcome composition of unique gRNAs across SCORE-SV windows. Any unique gRNA was flagged as “SV” if one or more clones were confirmed by WGS; otherwise, “non-SV.” (**C**) Editing outcome compositions across guide-donor pairs targeting 220 protein-coding genes stratified by SCORE-SV values: 0 to 0.15 (left); 0.15 to 0.5 (middle); 0.5 to 1.0 (right). Event counts (*y* axis) were normalized for repeated sampling of unique gRNA sequences (e.g., a single SV out of four clones with the same gRNA sequence was counted as 0.25). (**D**) Schematic guideline for high-accuracy CRISPR library creation. After filtering candidate oligos using SCORE-unedit, sites can be stratified by their error susceptibility (SCORE-SV) for differentiated engineering steps.

Setting a threshold of SV likelihood score greater than 0.15, we flagged 186 of the 1122 clones as SV prone, which captured 94.3% (83 of 88) of true SVs confirmed by WGS (Fig. 4A). Despite SCORE being trained only on data obtained with SpCas9, this threshold detected SVs equally well in both the SpCas9 and LbCas12a libraries (SpCas9: 10 of 11; LbCas12a: 73 of 77), suggesting that the SCORE predictions for SV formation are generalizable across different nucleases (Fig. 4B and fig. S16).

In addition to the 0.15 threshold, we noticed that an SV likelihood cutoff of 0.5 was able to distinguish sites with an intermediate risk for SVs from sites with a high risk (Fig. 4, B and C). This was consistent with HDR donor recruitment reducing SV incidence at sites with moderate SV scores between 0.3 and 0.5, but not at four sites with high SV scores >0.5 (Fig. 3C). Similar to SpCas9, LbCas12a targets showed an increasing SV rate with higher SV scores, albeit with an overall higher SV rate (Fig. 4B).

The ability of SCORE to stratify a CRISPR library based on unintended repair outcome profiles presents an effective strategy for producing high-quality variant libraries and reducing false-positive hits by prioritizing clonal isolation and validation of edits in SV-prone loci (Fig. 4D). For instance, targets with a low SV risk (0 to 0.15) are safe for pooled experiments as they are expected to yield predominantly correct edits. For targets with moderate SV risks (0.15 to 0.5), arraying the edited population followed by sequence validation enables generation of clonal populations devoid of undesired genotypes. High-risk SV sites (>0.5) are more challenging to edit and likely require screening of a high number of clones to obtain a correct edit. These targets should be held out at the design stage or flagged during analysis to mitigate false-positive phenotypic interpretations.

### The editable landscape of the synthetic yeast genome

The Sc2.0 synthetic yeast project involved the de novo design and synthesis of the 16 *S. cerevisiae* chromosomes (*36*, *37*). While based on the laboratory strain S288C reference genome, each chromosome was redesigned with major sequence changes to maximize genome stability and to enable previously unknown genomic functionalizations. These included the deletion of repetitive elements, introns, and tRNA genes, recoding of tandem repeats within and between open reading frames (ORFs) by using synonymous changes that minimize recombination potential, changing all TAG stop codons to TAA to free up the TAG codon for future noncanonical amino acid incorporation, and the introduction of loxPsym sites for the SCRaMbLE system by Cre-lox induction (*38*).

The Sc2.0 gene content is closely related to the S288C genome used in the development and validation of SCORE, which allowed us to ask how the SV-risk landscape was altered by the design features mentioned above. We hypothesized that the removal of repetitive elements should reduce the fraction of the genome prone to SV formation and enable editing at sites previously uneditable in the laboratory strain, including subtelomeric regions, loci with repetitive ORFs, and between tandemly repeated domains within ORFs. On the other hand, we reasoned that the inclusion of the 34-bp loxPsym sites could add potential for recombination, particularly large deletions, when situated close together.

**Fig. 5.**
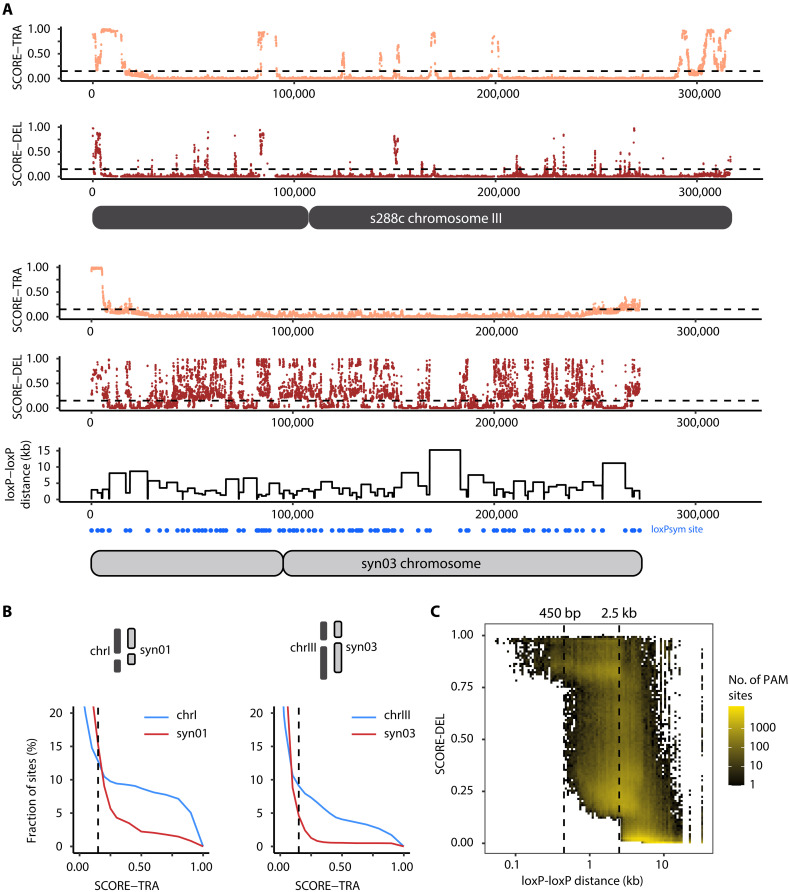
The editable landscape of the synthetic genome Sc2.0. (**A**) Predicted SCOREs for translocation (TRA) and deletion (DEL) formation across all unique NGG PAM sites on S288C chromosome III and its reengineered version in Sc2.0 (syn03). For the synthetic chromosome, the position of inserted loxPsym sites is shown in the bottom panel. The distance between consecutive loxPsym sites is shown as a line plot. (**B**) Percentage of targetable sites with a risk of inducing translocations (TRA) in chromosome I and III, and their reengineered version in Sc2.0. (**C**) Heatmap illustrating the relationship between predicted deletion score and loxP-loxP distances. The dotted lines at 450 bp and 2.5 kb show distinct jumps in the predicted deletion tendencies, such that all sites <450 bp and 2.5 kb apart have SCORE-DEL >0.75 and >0.15, respectively.

We applied SCORE to predict SV subtypes DEL and TRA across 859,529 NGG sites on the 16 synthetic Sc2.0 chromosomes. As expected, hotspots with high TRA scores in the native S288C genome were largely absent in their Sc2.0 counterparts (fig. S17). Chromosome III was the most affected, with five high-risk TRA regions in the chromosome body and one in the telomeric region removed in Sc2.0 (Fig. 5A). All synthetic chromosomes exhibited a lower fraction of high-risk TRA sites (TRA score > 0.5; fig. S18 and table S12), with chromosome I and III showing the most substantial decreases by 74.4 and 86.6% relative to the S288C genome (from 1332 to 325 TRA-risk sites in chromosome I, and from 938 to 114 sites in chromosome III) (Fig. 5B). While all chromosomes have similar lengths for the telomeric and subtelomeric regions, there is substantial variation in the chromosome arm length. Chromosomes I and III have the largest subtelomeric-to-arm length ratios in the yeast genome, due to their relatively short arms, resulting in the highest proportion of TRA-prone sites. On the other hand, the deletion likelihood map was markedly altered by the presence of the 34-bp Cre-loxP sites, with a negative correlation observed between adjacent loxP distances and the predicted deletion rate (Pearson’s *r* = −0.54, *P* < 2.2 × 10^−16^) (Fig. 5, A and C). It is not just the loxPsym sites that are problematic for Cas9 targeting, but the regions in between the sites themselves are rendered deletion prone despite containing unique target sites. Overall, the fraction of the Sc2.0 genome with high SCORE-TRA risk was reduced by half from 1.46 to 0.70%. Our analysis identified regions of the Sc2.0 genome that are likely to be refractory to downstream modifications by CRISPR nuclease-based approaches. This example highlights the value of SCORE in advancing future de novo genome sequence designs that minimize deletion and translocation risks (Fig. 5C).

## DISCUSSION

To improve the accuracy and fidelity of CRISPR editing for applications ranging from genome-wide screens to therapeutic editing, it is critical to characterize the spectrum and frequencies of unintended changes to the genome, from small-scale off-target point mutations to large-scale chromosome-level changes such as SVs. Furthermore, editing outcomes need to be assessed in diverse chromosomal contexts, as the endogenous DNA repair pathways responsible for generating various lesions in response to SSBs and DSBs can be locus-dependent (*39*–*41*). WGS of edited clones is the gold standard, yet a large-scale survey of thousands of sites is currently intractable in organisms with large complex genomes such as humans, where >500-fold more reads are needed to achieve the same sequencing depth as in yeast. In this study, we analyzed 1875 edited yeast genomes randomly sampled from a genome-wide library of natural variants to explore editing outcomes in an unbiased fashion. Our analysis revealed that the likelihood of SV formation upon CRISPR is not uniformly distributed across the yeast genome, but rather strongly enriched within a limited subset, within 4.8% of the genome, which exhibited frequent and recurrent SV formation (Fig. 1F). Notably, the risk of forming SVs persisted even in editing systems optimized for high editing efficiency (i.e., MAGESTIC v1.3 with >80% efficiency, fig. S3A) and is likely independent of the targeting and cleavage machinery, including optimizations to the guide and the Cas nuclease or with different nucleases such as LbCas12a (Fig. 4B). Our attempts to overcome SV formation with an improved donor template repair system using recruitment of retron-generated ssDNA donor templates and in vivo plasmid assembly (MAGESTIC 3.0) were successful only at a subset of SV-prone loci (Fig. 3), indicating that a fraction of the yeast genome is potentially off-limits to HDR-based editing approaches.

Our data suggest that SVs in yeast are driven primarily by HDR with endogenous homologies that can present themselves at a broad range of distances from target sites, from several tens to many thousands of base pairs. This is likely because the resection of broken DNA ends at the target site can extend up to thousands of bases, generating partial single-stranded DNA that is exposed to the homology search machinery, thereby enabling recombination to initiate far from the DSB (*42*). Consequently, regions affected by SVs can lie at considerable distances from repetitive elements yet still harbor large stretches of uniquely targeting gRNA sequences without any apparent off-target potential (fig. S19). This makes manual assessments of SV likelihoods based on the local sequence context intractable. To improve CRISPR editing outcomes, it is essential to consider multiple layers of genomic context and identify the optimal combination that maximizes editing efficiency while minimizing undesired outcomes. Achieving this requires not only modeling the relationship between individual genomic features and editing outcomes, but also the capacity to compare diverse feature combinations and determine the most favorable design and editing strategy. In this regard, our machine learning model SCORE provides quantitative measures to meet these challenges. SCORE-unedit enables the selection of high-efficiency guides by avoiding certain gRNA sequence compositions (e.g., long T stretches) and regions with low chromatin accessibility. Complementarily, SCORE-SV evaluates local and distal repetitive sequences at different context lengths to identify and avoid regions at high risk for SV formation. The genome-wide set of SCORE predictions is available in an interactive genome browser (https://apps.embl.de/score/) for the community to use for applications ranging from CRISPR editing of individual loci to genome-wide variant screens. Users can prioritize gRNAs with high predicted efficacy and low predicted SV likelihoods and implement additional steps to isolate correct edits in SV hotspots by using MAGESTIC 3.0 to boost template repair and by screening many clones (Fig. 4D). SCORE can also be used to improve variant screens by prioritizing SV-prone loci for additional validation steps.

The propensity for DSBs to induce chromosomal rearrangements and gene conversions close to the telomeric ends has previously been reported in budding yeast (*43*), mouse embryonic stem cells (*44*), and human cancer cells (*45*). Subtelomeric regions in eukaryotic chromosomes exhibit high sequence repetitiveness both within and between each other, a trait that may represent evolutionary adaptation to replication stress in these regions (*46*). The tendency toward nonreciprocal translocations in these regions in haploid yeast is consistent with the enrichment of loss of heterozygosity toward telomeric arms observed in diploid wild isolates across the yeast species (*47*), where it has been proposed to aid in the expression of recessive phenotypes and to increase allelic diversification (*47*). Cells may have evolved higher rates of HDR in subtelomeric regions via break-induced replication (BIR) in response to the greater prevalence of replication stress-induced DSB in these regions (*46*). Another factor contributing to the higher tendency for subtelomeric translocations over exogenous donor HDR in our assay may be due to subtelomeric regions exhibiting elevated levels of SSBs (*48*), which have been shown to improve HDR efficiency when occurring on endogenous chromosomal donors (*49*). Supporting this hypothesis, a study in *S. cerevisiae* reported higher survival rates of strains with DSBs near (sub)telomeric regions compared to intermediate chromosomal regions (*43*). Similarly, a study using a donor-free CRISPR system in *Yarrowia lipolytica* observed higher fitness of cells with gRNAs targeting subtelomeres (*50*), from which they concluded a lack of cutting activity at these sites. However, our and others’ investigations of repair outcomes (*43*) suggest that CRISPR nucleases cut efficiently near chromosome ends, but that DSBs in (sub)telomeric regions are readily resolved by repair with endogenous chromosomal donors, leading to SV formation and cell survival. Overall, understanding the genome-wide distribution of repair outcomes, informed by genomic contexts, would enable editing strategies to overcome or at least manage the risk of erroneous outcomes. Efforts to modulate these pathways toward preferred outcomes have included chromatin remodeling (*51*), mismatch repair manipulation (*52*), or enhanced donor HDR [e.g., MAGESTIC 3.0 (*35*)].

The consideration of long-range genomic context is a major difference between SCORE-SV and previous computational models, which so far have been limited to associating local sequence contexts with editing efficiency [e.g., for Cas9 nuclease (*12*), base editors (*53*), and prime editors (*54*)] and for predicting smaller-scale unintended on-target outcomes, such as NHEJ indels (*14*, *55*), bystander edits (*53*), and pegRNA scaffold insertions. While it is well-established that nuclease efficiency and NHEJ-induced indels are influenced by the local sequence context (*12*, *14*), our findings highlight the importance of long-range sequence features, chromatin state, and chromosomal context (e.g., proximity to telomeres). Models trained on editing outcomes from reporters outside of the endogenous genomic context (e.g., those based on editing artificial constructs or endogenous target sites removed from their native locus) are likely to fall short in predicting large-scale events, which may occur only in specific chromosomal regions. Therefore, it is crucial to have methods capable of capturing both small-scale and large-scale changes. Various non-WGS methods have been developed to characterize particular classes of SVs at target sites in human cells, including anchor-based Tn5 approaches to capture translocations that preserve one side of the target site (e.g., PEM-seq) (*56*), Southern blotting or use of panels of primers flanking the target site combined with long-read sequencing to capture mid-size (hundreds of base pairs to several kilobases) deletions (*9*) or unanticipated insertions (*57*), and single-cell RNA sequencing to infer large deletions or chromosomal arm loss (*6*). Each of these methods, however, has limitations and may fail to detect certain types of SVs at target sites. Complementary to these approaches are copy number analysis by droplet digital PCR (*58*) and loss of fluorescence from single-copy insertions of GFP reporters at or near target sites to infer target site disruption (*26*). While these assays provide valuable insights, they are difficult to scale across multiple genomic sites and offer only limited information about the sequence at the SV breakpoints, which is important for developing predictive models for SV formation.

One limitation of the current study is that the SV landscape was characterized in a single laboratory strain background of *S. cerevisiae* using a single CRISPR-Cas9–based editing system (MAGESTIC). Because our predictive model, SCORE, was trained on this dataset, it may be biased toward the DNA repair preferences of this strain under these specific conditions (i.e., donor recruitment). However, it is likely that the model can be generalized to other systems with similar DNA repair profiles, specifically those with high proficiency in HDR over error-prone pathways such as MMEJ. Supporting this, we observed that SV breakpoints in 142 natural yeast isolates (*31*) frequently overlapped with SV-prone sites identified in our laboratory strain (fig. S14), suggesting that these strain backgrounds may share similar vulnerabilities to SV formation upon natural or artificially introduced DSBs (i.e., by the CRISPR-Cas system). For organisms more distantly related to *S. cerevisiae* (e.g., human), we anticipate that a similar set of genomic features used in SCORE (i.e., local and global sequences and chromatin context) could be applied to predict SV likelihoods, at least those driven by HDR-mediated processes. However, the model would need to be retrained to capture the species-specific DNA repair preferences. The observation that a larger variety of SV types can arise in human cells (e.g., MMEJ-mediated deletions, NHEJ-mediated translocations and inversions, and chromosome arm truncations) make both the unbiased detection and modeling more challenging than in the yeast system. In addition, SVs in yeast appear to be confined to a specific subset of the genome where they occur systematically at a very high rate, such that most of the genome exhibits little-to-no detectable SV formation. In contrast, studies on mammalian systems have reported large deletions across a wide range of frequencies for all target sites thus far examined, ranging from 0.2 to 17.5%, depending on the chromosomal context and cell type (*9*). These deletions appear to be largely driven by microhomologies that cannot be readily predicted by sequence context alone (*25*). Key questions remain unanswered about whether SV rates vary across the genome and whether there are SV hotspots in mammalian genomes as we found in yeast. It is likely that existing studies have not examined a sufficiently large or representative set of genomic sites for building predictive models for editing outcomes. Future efforts should focus on developing scalable approaches to map the wide range of possible SVs observed in mammalian systems.

## MATERIALS AND METHODS

### Yeast strains

The BY-derived haploid strain DHY214 was used as the base strain for editing with MAGESTIC, with additional modifications on the landing pad of genome-integrated barcodes depending on the editing system version. A complete list of the genomic landing pads and plasmid backbones is shown in table S10.

### Genome editing and barcoding

We performed highly multiplexed and parallel editing of the designed variants using published protocols (*16*), with adjustments depending on the version of the editing system. Libraries of gRNA and donor DNA pairs were designed to incorporate polymorphisms present in strains RM11 or SK1 relative to the S288C reference genome. We tested the performance of three successive improvements to our MAGESTIC editing system, named MAGESTIC v1.1, v1.2, and v1.3 (*n* = 81, 471, and 1323, respectively), for the installation of several natural variant libraries. Compared with MAGESTIC v1.0 (*n* = 52), the newer versions were optimized by replacing the galactose-inducible *GAL1* promoter with the constitutive *TEF1* promoter for Cas9 expression (v1.1), replacing the tRNA(Tyr)-HDV promoter with the *SNR52* promoter for gRNA expression (v1.2), and adding a tetracycline-inducible RPR1-TetO promoter (*59*) to drive expression of the gRNA used for the barcoding step to temporally separate editing from barcoding (v1.3). A barcode reference table was constructed for each library by amplifying the guide-donor-barcode region from the plasmid libraries at the first stage of cloning (before inserting the sgRNA scaffold and markers in between the gRNA and the donor) with indexed Illumina primers.

### Recombinase-directed indexing

Edited and barcoded yeast library pools were plated from glycerol stocks onto YPD plates and grown for 2 days at 30°C. Single colonies were randomly selected and arrayed into 384-well plates using an automated colony picking robot (RapidPick Lite, Hudson Robotics). After an overnight growth at 30°C, four 384-well plates were combined into a 1536-well array using a Singer RoToR HDA (Singer Instruments, UK) and used for REDI. Briefly, the *MAT*α haploid strains were mated with barcoded *MAT***a** REDI strains arrayed in 1536-well format using the Singer RoToR. Through Cre-induced recombination of the barcode locus (induced by pinning on SC-URA + GAL plates), a single allele in the diploid strain contains both the editing barcode (informing on the installed variant) from the *MAT*α strain, and the 1536-well plate index barcode from the *MAT***a** parent. To determine the position of specific edited variants on the array, the mated cells were washed off the plate, genomic DNA was extracted with a kit (Epicentre), and the barcode locus was amplified via PCR using indexed Illumina primers. The resulting reads (sequenced on Illumina MiSeq system, 2 × 150 bp) were merged using BBMerge (*60*), plate positions were identified via mapping of index barcodes to the set of available index barcodes (only perfect matches considered), and variants were identified by mapping the MAGESTIC barcode to the barcode reference table.

For clones generated with the v1.3 system REDI batch 2 (table S1), two separate PCRs were necessary to map MAGESTIC barcodes to index barcodes due to a different structure of the barcode locus. Here, the first amplicon linked the MAGESTIC barcode and the donor DNA fragment, whereas the second amplicon linked the index barcode and the donor DNA. MAGESTIC barcodes were mapped to index barcodes by aligning donors from each amplicon to the set of designed donors using BLAST+ (*61*).

The selected set of variants was picked with a colony picker using the index barcode information and rearrayed into 96-well microwell plates, grown overnight, and stored as glycerol stocks. Selection criteria included that each variant be represented with a maximum of four barcodes, without sequence errors in the gRNA sequences (batches 1 and 2), and without sequence errors in the 60-nucleotide (nt) region centered on the target mutation in the donor DNA region (batch 2). In batch 1, we allowed sequence errors anywhere in the donor DNA to assess the impact of synthetic donor errors on editing efficiency and rate of incorporation of the donor errors into the genomic edits

### WGS library construction

Selected strains were inoculated into 100 μl of YPAD from glycerol stock and grown overnight at 30°C, 800 rpm. WGS libraries were prepared using our extraction-free method (*18*) and, following the protocol described in File S1, adapted to a 96-well format. We used the triple mutant homemade Tn5 and the protocol version with Proteinase K treatment for nucleosome dissociation. Magnetic bead cleanup after Proteinase K treatment was performed on a liquid handling robot (Biomek Fxp, Beckman Coulter), while samples from the entire plate were pooled for cleanup after the PCR step. Samples were sequenced on an Illumina NextSeq platform using 2 × 150-bp reads, at an estimated coverage of 5×. As the site of the target edit is known by virtue of the MAGESTIC barcode, we reasoned that such low coverage is sufficient to detect the editing outcome at the target site.

### Read data processing and editing outcome identification

Raw reads in FASTQ format were processed by trimming off adapter sequences using cutadapt (*62*) and mapped to the sacCer3.0 reference genome (http://sgd-archive.yeastgenome.org/sequence/S288C_reference/genome_releases/S288C_reference_genome_R64-2-1_20150113.tgz) using bwa-mem (*63*). The mapped BAM (Binary Alignment/Map) files were sorted and marked for PCR duplicates, and the base quality scores were recalibrated using GATK4 (*64*).

Following successful MAGESTIC barcoding, a portion of the guide-donor plasmid, including the full-length donor template, integrates into the genome at the landing pad. Reads overlapping the barcode region can be partially mapped to the target site due to sequence similarity in the gRNA and donor region, leading to false discovery of intended edits. To prevent this, a custom script was implemented to filter out reads containing chimeric junctions, which suggest origins from barcode sequences or residual plasmid fragments in the WGS libraries. Last, variant calling was performed using GATK4 HaplotypeCaller (SNV and indel) and SvABA (*65*) (SV), followed by downstream analysis to define different categories of editing outcomes.

To distinguish intended from unintended variants at the target sites and account for incomplete editing of MNVs, we developed an annotation method based on edit distances (Levenshtein). A variant is classified as intended if its addition to a wild-type sequence reduces the edit distance to the donor template; otherwise, it is classified as unintended. This approach also characterizes editing status of oligo synthesis errors by treating them as additional designed variants on donor templates. A schematic of the variant definition is shown in figs. S20 and S21.

### SV identification and annotation

Short read-based algorithms for SV detection are known to be suboptimal under certain conditions (e.g., repetitive or highly mutated regions, low sequencing depth) that lead to low recall and high false-positive rates (*66*). In this study, we used a three-way cross-validation approach to ensure accurate SV annotation across diverse target contexts. Potential SVs were preselected by three methods independently: (i) on-target deletions or translocations identified by SvABA; (ii) unintended SNVs or indels within 200 bp up- or downstream of the target site identified by GATK4 HaplotypeCaller; and (iii) copy number loss detected by reduced read coverage at the target site. Subsequently, regions containing at least one of these signatures were visually inspected and compared to unedited controls in the IGV (Integrative Genomics Viewer) (*67*) to confirm true SVs.

Copy number changes at target sites were assessed using a custom script. Binned coverage metrics were computed for each WGS sample with a 250-bp bin size, resulting in an *m* × *n* matrix, where *m* is the number of samples and *n* is the number of bins. Raw read counts were normalized for library size variation using DESeq2 (*68*), yielding a matrix $\underline{X}=\left(x_{1,1}\cdots x_{1,m}\vdots \ddots \vdots x_{n,1}\cdots x_{n,m}\right)$ , where *x_i,j_* denotes the normalized read count in bin *i* of sample *j*. These counts were then converted to ranks across bins: $\begin{array}{c} R_{i,j}= \end{array}$  $\begin{array}{c} \frac{\text{rank}\;\text{of}\;x_{i,j}\;\text{in}\;\left(x_{1,j},x_{2,j},\ldots x_{n,j}\right)}{n},\left(i=1,2\ldots n\right) \end{array}$ . The inverse of *R_i,j_* reflects the likelihood of a region being under-sequenced, indicating potential copy number loss. To rule out background variation in read depth (e.g., from Tn5 insertion bias or GC content), the “rank of rank” (RoR) was computed to normalize ranks across samples: ${\text{RoR}}_{i,j}=\frac{\text{rank}\;\text{of}\;R_{i,j}\;\text{in}\;\left(R_{i,1},R_{i,2},\ldots R_{i,m}\right)}{m}$ , (j=1,2…m) . Bins with RoR < 0.1 were considered as signals of copy number loss.

To identify corresponding regions involved in repair at SV loci, reads containing unexpected variants relative to the target site (SNVs, soft-clipped sequences) were aligned against the reference genome using blastn (*61*) (table S2). On the basis of the location of detected hits, the variants were classified as deletions or translocations. In case of translocations, the alignment of the template regions was inspected in IGV to search for potential reciprocal translocations.

### Analysis of off-target mutations

Genomic Call Variant Format (GVCF) files were generated from the BAM files of 2260 genomes and combined into a single file using the “GenotypeGVCFs” command of GATK4. Variants in the combined VCF (Call Variant Format) file were filtered to exclude low-quality calls with the expression “QD < 2.0 || FS > 60.0 || MQ < 40.0 || SOR > 3.0 || MQRankSum < −12.5 || ReadPosRankSum < −8.0.” Additional filters were applied to exclude the following: (i) variant loci with missing genotype “.” in >50% of samples; (ii) samples with missing genotypes “.” in >50% of loci; and (iii) variants shared across multiple samples with different gRNA sequences. For the remaining variant loci, genomic sequences ±25 bp around the PAM site were extracted and aligned to the gRNA sequence (20 nt + NGG) using the semiglobal algorithm (gaps are allowed at the beginning or ends of the shorter gRNA sequence but not the longer genomic sequence). Alignments were performed in both forward and reverse direction, with the best hit retained per locus. The edit distance between the best hit and the gRNA sequence is depicted in fig. S4B.

### Quantifying genomic repetitiveness using local and distal repetitiveness indexes (*I*_LR_, *I*_GR_)

For any given genomic location, the local sequence repetitiveness score *I*_LR_ [as originally defined and termed as *I*_r_ (*27*)] was calculated for a defined window size (50 bp, 100 bp, 500 bp, 1 kb, 5 kb, or 10 kb) centered at the target site (defined as the first nucleotide of the PAM sequence). *I*_LR_ describes the complexity of a given DNA sequence by the length of the shortest unique substring at each base position. Inspired by this concept, we developed a custom script to compute *I*_GR_, which quantifies the global repetitiveness through mapping the shortest unique substrings between a target region (50 bp, 100 bp, 500 bp, 1 kb, 5 kb, or 10 kb) and the rest of the genome. The script is embedded in the feature table preparation step of the SCORE workflow (https://github.com/shli-embl/MAGESTIC-SCORE). Tandem repeat length was defined as the longest homologous sequence (with a maximum of one mismatch) between the left and right PAM-adjacent regions (with 25 bp, 50 bp, 250 bp, 500 bp, 2.5 kb, or 5 kb per arm), excluding matches in opposite strand directions (i.e., excluding inverted repeats).

### Predictive model construction

#### Featurization

Three sets of genomic features describing the target-specific context and two technical variables (designed variant type and editing system) were included in the training of SCORE (table S5) to regress out confounding effects. Feature subset 1 includes all metrics relevant to the local sequence context of a targeted site (20 bp gRNA sequence ±5 bp, GC content, and T homopolymer score). Feature subset 2 includes chromatin accessibility and transcriptional and histone modification status. Feature subset 3 includes metrics of repetitiveness and target location on chromosome (distance to telomere and centromere). Feature table preparation is detailed in Supplementary Text. Features applied for each SCORE predictor were determined based on evaluating gradient-boosted decision tree (GBDT) performance across all six combinations of the three feature subsets (fig. S10).

#### Hyperparameter tuning

To determine the optimal model for our task, we conducted a pilot analysis comparing four algorithms: (i) GBDT, (ii) random forest, (iii) L1-regularized logistic regression, and (iv) L2-regularized logistic regression. A grid search was performed for hyperparameter tuning. Model performance was evaluated using repeated *k*-fold cross validation (repeat = 5, *k* = 3). The selection criteria included AUROC, AUPRC, and F1 score. Algorithm-specific hyperparameters included the following: (i) number of trees, learning rate, and interaction depth for GBDT; (ii) number of trees and random variables at each split (mtry) for random forest; and (iii) the penalizing factor λ for L1- and L2-regularized regressions.

We tested a set of strategies to address class imbalance in the training data (more correctly edited loci, fewer SVs, and unedited sites), including (i) random undersampling (RUS) of the majority class, (ii) random oversampling (ROS) of the minority class, (iii) Synthetic Minority Oversampling Technique (SMOTE) (*69*) oversampling of the minority class, and (iv) ADASYN (Adaptive Synthetic Sampling approach) (*70*) oversampling of the minority class. Undersampling was treated as a hyperparameter with levels of 10, 20, 50, and 100% of the original majority class size. Each level was combined with one of the oversampling techniques (RUS + ROS, RUS + SMOTE, and RUS + ADASYN) or applied alone (RUS only) to achieve a 1:1 class ratio in the training set. These adjustments were applied only to the training data, while validation splits (within cross-validation) remained unchanged for evaluation. The optimal hyperparameter combination is outlined in table S6. Final predictors were trained by using the complete dataset without a separate validation split. The output predicted value was min-max normalized to be displayed in the range from 0 to 1, i.e., SCORE-SV = (model output − SCORE-SV_min_)/(SCORE-SV_max_ − SCORE-SV_min_), where _min_ and _max_ correspond to minimum and maximum values observed on the reference genome.

### Feature importance assessment

Feature importance was defined based on permutations. Briefly, values of individual or grouped features were randomly shuffled across samples, and the difference in AUROC before and after shuffling quantified the feature’s contribution to model performance. Each test included 1000 permutations. The resulting AUROC values were used to determine 90% confidence intervals.

### Functional annotations of genomic regions

Annotations of protein-coding, noncoding (tRNA or ncRNA), telomeric, and transposable element regions were extracted from the S288C gene annotation file (as part of the downloaded reference genome files at http://sgd-archive.yeastgenome.org/sequence/S288C_reference/genome_releases/S288C_reference_genome_R64-2-1_20150113.tgz). These elements were mapped to 873,419 NGG sites across the S288C genome. NGG sites not overlapping with any of the elements were categorized as intergenic.

Sites overlapping protein-coding genes were further stratified based on essentiality. The list of essential genes was downloaded from yeastMine (via the AllianceMine website: https://alliancegenome.org/bluegenes/alliancemine) using the “Retrieve all phenotypes for all genes” panel, followed by “View -> View Rows” and filtering for entries where “Phenotype Observable” equals “inviable.” TATA box containing genes, known to enrich for stress responsive genes, were extracted from a previous publication (*29*) (see table S2 in the noted reference).

### SV hotspot identification

An SV hotspot was defined as a cluster of NGG PAM sites in close proximity, which exhibited a high SV likelihood predicted by SCORE. Specifically, adjacent NGG PAMs with SCORE-SV > 0.15 on either chromosomal strand were grouped into clusters. Clusters containing fewer than 10 consecutive SV signals were excluded. Last, the remaining clusters within 200 bp of each other were merged to form SV hotspots, as listed in table S8.

### Natural SV breakpoint analysis

The list of 4809 natural SVs was downloaded from the original publication (*31*) as a supplementary table. SV-type annotations were retained as reported in the source, comprising six categories: contraction, duplication, deletion, insertion, inversion, and translocation. For each SV, both the start and end coordinates were considered as distinct SV breakpoints, resulting in a total of 9618 breakpoints for downstream analysis.

For each SV breakpoint, we defined a ±1050 bp window (i.e., from −1050 bp to +1050 bp), where all NGG PAM sites with available predicted SV scores were extracted. These sites were then grouped into 100-bp bins based on their relative distance from the SV breakpoints.

As our prediction scores have prefiltered PAM sites associated with nonunique guide sequences (i.e., ones that would lead to multiple cuts in the genome), these excluded positions did not contribute to the visualization of SV scores. Their distribution across bins was instead analyzed separately by quantifying their fraction in each 100-bp bin (i.e., fig. S14, bottom panel).

### MAGESTIC 3.0 editing system for SV-prone targets

We selected eight pairs of gRNA and donor templates causing different types of SVs (DEL, FR, and TR) and residing at diverse chromosomal locations (table S9). For each target site, the same guide was used as that used in the natural variant library. To prevent the possibility of the guide re-cleaving the target site after editing and potentially leading to post-edit SV formation, multiple SNVs were engineered in the PAM-proximal region of the guide. The guide-donor oligos were designed as 200-mers and cloned into plasmid backbones as described (*35*). For the nonplasmid assembly versions, the cloned plasmids were transformed directly into yeast (table S10). For guide-donor plasmid assembly in yeast, the guide-donor plasmids were first digested by I-Sce I in vitro and the guide-donor backbones purified by gel extraction. Two hundred nanograms of the resulting linearized guide-donor vectors was transformed with 200 ng of pF811 insert (for I-Sce I and SaCas9-mediated barcoding) (table S10).

All yeast transformations were pelleted in PCR strips, resuspended in 50 μl of H_2_O, and 20 μl was transferred to 480 μl of complete synthetic medium lacking uracil, histidine, and leucine (CSM-Ura-His-Leu) in a deep well plate to select for maintenance of the Cas9 plasmid, uptake of the guide-donor editing vectors, and prevention of premature barcoding, respectively. After 2 days of growth at 30°C with shaking at 800 rpm, 16 μl from this first outgrowth was transferred to 484 μl of CSM-Ura-His-Leu for a second outgrowth (32-fold dilution to enable five generations). The next day, 16 μl was transferred to 484 μl of CSM-Ura-His + galactose (instead of glucose) to initiate barcoding and guide-donor plasmid self-destruction. For the MAGESTIC 3.0 SaCas9-barcoding system, anhydrotetracycline (1 μg/ml) was also added to induce SaCas9 and its guide X1. This was repeated for a second barcoding outgrowth. Last, 16 μl of the second barcoding outgrowths was inoculated into 484 μl of CSM-His + 300 μM 5-fluorocytosine to counter-select for any cells harboring guide-donor plasmids while simultaneously selecting for successful integration of the *HIS3*-barcode–containing inserts at the chromosomal barcode (REDI) locus. Genomic DNA from these cultures was prepped using the MasterPure yeast DNA extraction kit (LGC Biosearch Technologies, Inc). WGS libraries were prepared with the Illumina DNA prep kit (which uses Bead-linked transposons). The libraries were sequenced on the Illumina NovaSeq S4 lane by 2 × 151-bp reads by Novogene USA (Davis, CA).

### Bulk WGS analysis

Raw sequencing reads were processed and mapped to a customized S288C reference genome, incorporating several additional sequences: (i) guide-donor plasmid, (ii) genomic landing pad of the MAGESTIC barcode cassette, and (iii) editing cassette plasmid. Per-base read counts for each of the nine regions per sample were generated using “samtools depth,” including unedited sites as control for downstream analysis.

Raw read counts were normalized for library size by dividing by the mean read depth per sample. Normalized counts were calculated for both unedited samples (with different gRNAs from the inspected site) and the edited sample. Normalized coverage was indicated by the ratio of normalized counts in the edited sample to that in the unedited control (averaged across all control samples). This per-base coverage was binned using a sliding window of length adjusted based on region size (window size = region size/25; step size = region size/50). Coverage reduction (Δcoverage) was calculated as (1 – normalized coverage), reflecting the fraction of bulk samples with SVs.

To determine the composition of correct edits and unedited fractions, reads overlapping the targeted variant (or its wild-type sequence) were extracted and counted. For each sample, the fraction of SV (Δcoverage) was subtracted from 1, and the remaining fraction was partitioned into edited and unedited fractions according to the ratio of read counts for edited and wild-type alleles.

### Creation of independent CRISPR-Cas9 and CRISPR-Cas12a libraries using MAGESTIC3.0

An independent library of guide-donor plasmids was generated for MAGESTIC 3.0-based editing of all targetable variants in 112 previously mapped quantitative trait loci with SpCas9, LbCas12a, and impLbCas12a nucleases, as described in a previous study (*35*). After editing and barcoding outgrowths, cells were plated for growth as single colonies onto CSM-HIS+5FOA (1 mg/ml) agar and were arrayed with a PIXL robotic colony picker (Singer Instruments, UK). The integrated genomic barcodes for each clone were identified using REDI (as described above). Clones with barcodes corresponding to sequence-perfect guide and donor sequences were identified for a rearray step, followed by stamping with the ROTOR (Singer Instruments, UK) [growth in 100 μl of YPD + G418 (100 μg/ml) 96-well format for genomic DNA extractions and −80°C archival in 384-well plates filled with 60 μl of YPD + G418 (100 μg/ml) +15% glycerol].

The integrated genomic barcode per clone was extracted from WGS data and mapped to a reference table to identify corresponding gRNA and donor DNA sequences. Clones without barcode information or with ambiguous barcodes were excluded. For the remaining 1122 clones, raw read processing, mapping, and outcome calling were conducted as described above for the 2260 clones, with modification to the reference genome to include variants specific to the editing base strain version used in this editing experiment.

For the CRISPR-Cas9 library, no edit, SV, DEL, and TRA prediction scores were extracted from the precomputed table available on the SCORE app website (https://apps.embl.de/score/). Predictions for the CRISPR-Cas12a library were performed using our workflow described above, with input coordinates adjusted to align with the format used for Cas9 datasets.

### Mapping SV-prone sites throughout the Sc2.0 genome

Sc2.0 genome sequences and annotation files were downloaded from the project website (https://syntheticyeast.github.io/sc2-0/data/). The loxP site distance was defined as the base pair distance between the two adjacent loxP sites upstream and downstream of the analyzed NGG site. SCORE predictions for SV, DEL, and TRA were generated for all NGGs in the 16 synthetic chromosomes using the models trained on the 1875 WGS datasets.

## Supplementary Materials

### The PDF file includes:

Supplementary Text Figs. S1 to S21 Legends for tables S1 to S12 References
