# Supplementary material for: The editable landscape of the yeast genome reveals hotspots of structural variant formation
Source: Sci Adv. 2025 Oct 31;11(44):eady9875. doi: 10.1126/sciadv.ady9875 (PMC12577706; doi:10.1126/sciadv.ady9875)
Supplement: Supplementary file 1 — Supplementary Text Figs. S1 to S21 Legends for tables S1 to S12 References [file sciadv.ady9875_sm.pdf]

Science Advances
AAAS

Supplementary Materials for

The editable landscape of the yeast genome reveals hotspots of structural variant formation

Shengdi Li *et al.*

Corresponding author: Lars M. Steinmetz, lars.steinmetz@stanford.edu

Sci. Adv. **11**, eady9875 (2025)
DOI: 10.1126/sciadv.ady9875

The PDF file includes:

Supplementary Text
Figs. S1 to S21
Legends for tables S1 to S12
References

Other Supplementary Material for this manuscript includes the following:

Tables S1 to S12

Supplementary Text

Investigation of incomplete edits in 2,260 genomes

The variant library used in the present study includes multi-nucleotide variants (MNVs). An MNV consists of multiple single variants spanning the template regions. In-between adjacent single variants are sequences identical between the genome and the donor template. As a potential result of template switching during target site repair, imperfect installation of MNVs may arise and become detectable by WGS.

To study the relationship between MNV format and installation outcomes, we sought to expand the MNV range by treating oligo synthesis errors as additional “designed” variants on the donor template. During the creation of a MAGESTIC library, a reference table is generated by sequencing the oligo library, capturing synthesis-derived sequence errors. Inclusion of these synthesis errors in the analysis allows for investigating incomplete edits across a diverse range of variant-variant distances, from 1 bp to >50 bp.

The MNVs and synthesis errors were converted into a list of neighboring single-variant-to-variant pairs. Each variant pair can be categorized according to its editing outcome: co-incorporated, only one variant incorporated, or neither variant incorporated. A workflow for comparing edit distances between WGS sequences, the reference genome and the designed donor template sequence was used for automatic annotation of editing outcomes of MNVs (**fig. S21**). For each variant pair, we also annotated the length of identical sequence, or inter-variant homology, and correlated it with editing outcome. This correlation analysis revealed that longer inter-variant homology leads to more frequent incomplete edits (**fig. S3C**).

Reproducibility of SV formation upon CRISPR/Cas9 editing at four sites

The largest WGS dataset generated in the present study (of 2,260 clones) is a collection of random sampling from a mixed cell pool of diverse target loci. As a consequence, the percentage of SV formation estimated represents a pool-level average across many loci (3.8% for MAGESTIC v1.3 and 7.7% for MAGESTIC v1.1). This frequency can either be due to a high rate of SV formation in a few targets (many cells with the same gRNA and repair template will form an SV), or a low rate across numerous targets (SV events commonly occur at many targets but only in a small fraction of cells with the same gRNA and repair template).

To distinguish between these scenarios, we cloned and re-transformed guide-donor plasmids for four of the targets flagged as SV events, and performed WGS on 78-166 clones for each target. The incidence of SVs was consistently high at all four tested loci, ranging from 71.1% to 97.6% (**Fig. 1F)**, indicating that SV occurrence for a specific target, gRNA and repair template is highly reproducible. However, we observed extensive heterogeneity in the recombination tracts of DELs and TRAs, and thus in the exact genotype of the resulting strain, for any individual target (**fig. S6C**). This heterogeneity likely arises from the broad, discontinuous homology between the target site and the template region. In fragmental replacements, for example, the start and end points of the replaced are determined by the extent of DNA end resection at both telomeric and centromeric sides of the Cas9 cut site. Taken together, these results revealed a highly context-dependent distribution of reproducible Cas9-induced SVs in those “difficult-to-edit” genomic regions.

Feature table preparation

A pipeline for extracting annotations for a given list of genomic positions (the PAM sites of interest) is available at our GitHub repository as a Snakemake (*71*) workflow (https://github.com/shli-embl/MAGESTIC-SCORE). The annotation files were processed from publicly available resources or the reference genome sequence as described below.

ATAC-seq data for DNA accessibility: The raw sequencing reads from a previous ATAC-seq experiment (*72*) were downloaded and aligned to the sacCer3.0 genome. The resulting BAM files were merged and processed using "pyatac ins –bam" from the NucleoATAC tool (*72*) to determine per-base transposon insertion frequency. A 50-bp window around the PAM site was used to calculate the mean insertion frequency (log scale), indicating DNA accessibility at the site of cleavage.

ChIP-seq data for 26 types of histone modifications: Raw data files for 26 histone modifications, along with input controls, were obtained from a previous publication (*73*). As the original study involved a time course of stress responses, only the untreated data point (time 0) was analyzed. BigWig files were converted to Wig format, and signal intensities were then binned using a 100bp sliding window with step size of 50 bp. For each target site, the mean signal intensity of two 100bp windows overlapping the PAM site was computed. Histone modification levels were expressed as $\log_2(\frac{\text{signal intensity}+25}{\text{input intensity} + 25})$, with 25 as a pseudo count.

Strand-specific RNA-seq: Data of wild-type samples with poly-A enrichment were downloaded from published work (*74*) and BAM files of two separate strands were generated using "samtools view -bf 16" and "samtools view -bF 16", respectively. Read depths were calculated using "samtools depth". A 50-bp window centered at the PAM site was used for extracting mean transcript level per strand, and then annotated according to the orientation relative to the PAM.

The repetitiveness indexes (I_{LR}, I_{GR}, TRL), distance to chromosomal ends, GC content and T homopolymer score were calculated using our in-house scripts (https://github.com/shli-embl/MAGESTIC-SCORE).

Editing system (v1.1/v1.2/v1.3) and variant type (SNV/indel/MNV) information was one-hot coded in the feature table for downstream processing.

Supplementary Figures

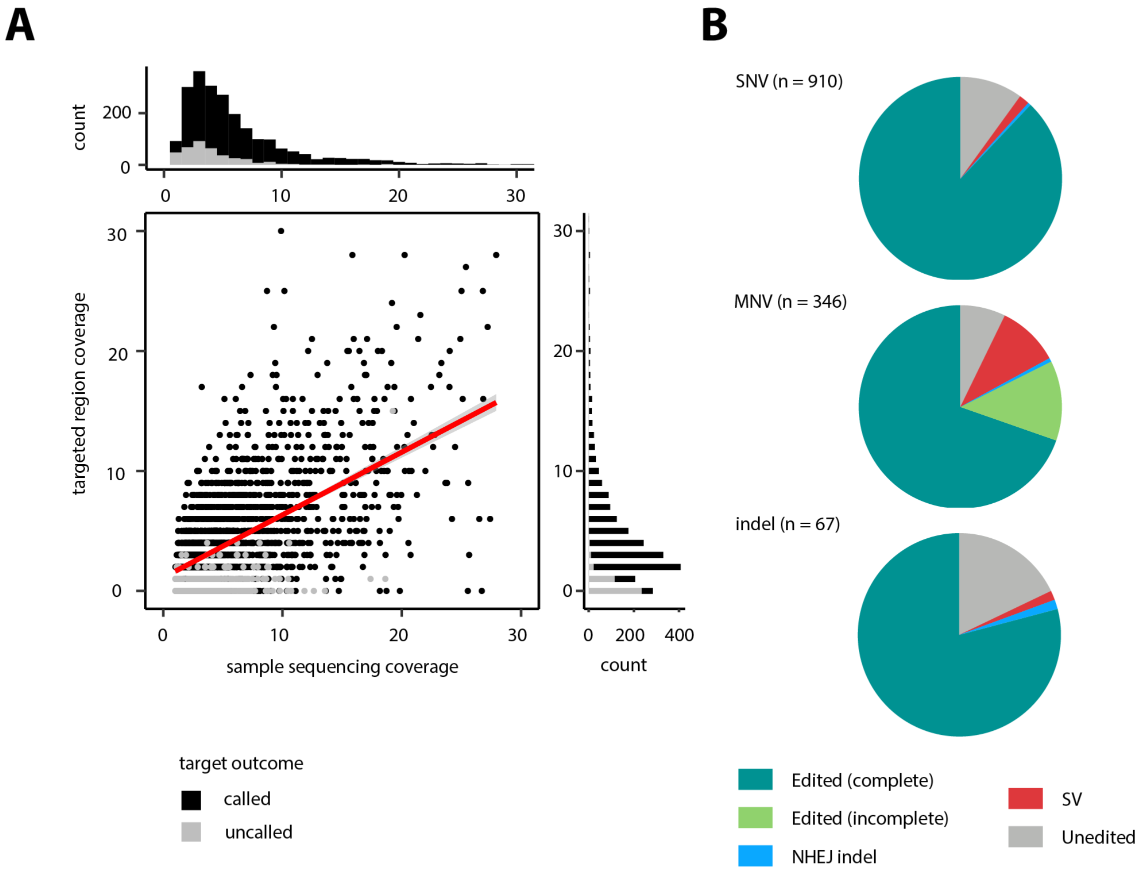

Figure S1. Whole-genome sequencing identifies editing outcomes across 1,875 clones
(**A**) Sequencing coverage at the target site and across the entire genome for the 2,260 target sites analyzed, with black and gray dots indicating called (1,875) and uncalled (385) target genotypes, respectively. Histograms show the distribution of event counts across different sequencing depths. (**B**) Distribution of editing outcomes for different types of designed variants across 1,323 clones using MAGESTIC v1.3 editing system (**Materials and Methods**). SV: Structural Variants; NHEJ: Non-Homologous End Joining induced SNVs or indels.

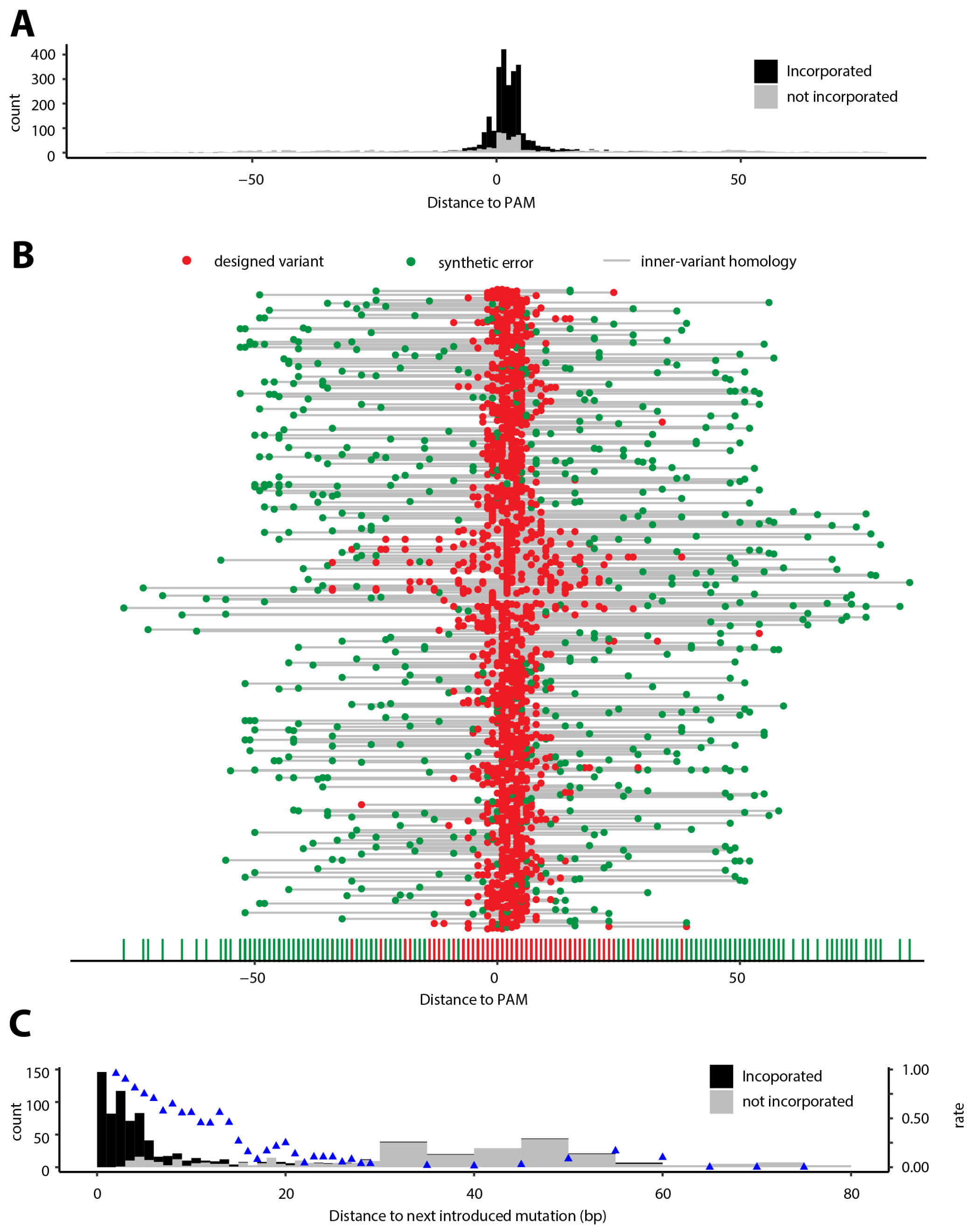

Figure S2. Incomplete editing is dependent on sub-variant distribution on the donor template

(**A**) The distribution of variant position on donor template and installation outcome. Coordinates are standardized relative to the PAM (from -2 to 0) and the gRNA seed region (from +1 to +20). (**B**) The distribution of synthesis errors on donor templates, excluding error-free templates. (**C**) The number of variant pairs was counted for each bin of distance to the next introduced mutation (bp) and according to the installation outcome. Variants with a distance >30bp to the next introduced mutations were grouped into 5-bp bins (e.g. 30-35, 35-40). Blue triangles represent the incorporation rate of a given distance by averaging the values from +1 to -1 bins relative to the tested bin (e.g. rate for distance = 10 is the mean of 9-11, distance = 40 is the mean of 35-45).

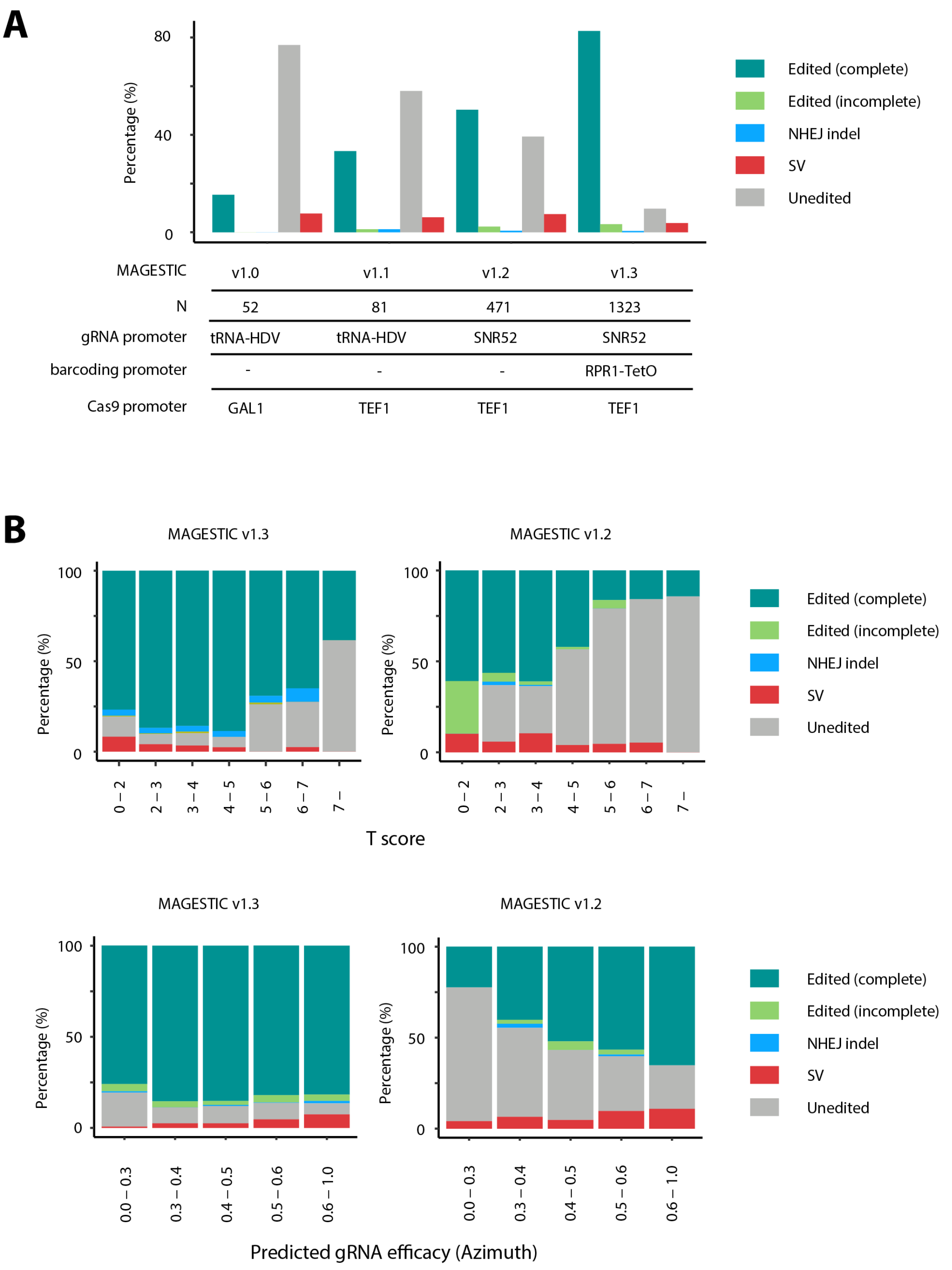

Figure S3. Distribution of editing outcomes with MAGESTIC editing systems v1.0 to v1.3
(**A**) Editing outcomes identified by WGS are shown for four datasets with different MAGESTIC versions (**Materials and Methods**). MAGESTIC v1.0 refers to the original editing system published previously (**Supplementary Text**). MAGESTIC v1.1 to v1.3 are systems used in the present study for the sequencing of 2,260 clones. (**B**) Comparison of editing outcomes between v1.2 and v1.3, stratified by T homopolymer scores (upper panel) or gRNA efficacy predicted by Azimuth score[6] (bottom panel).

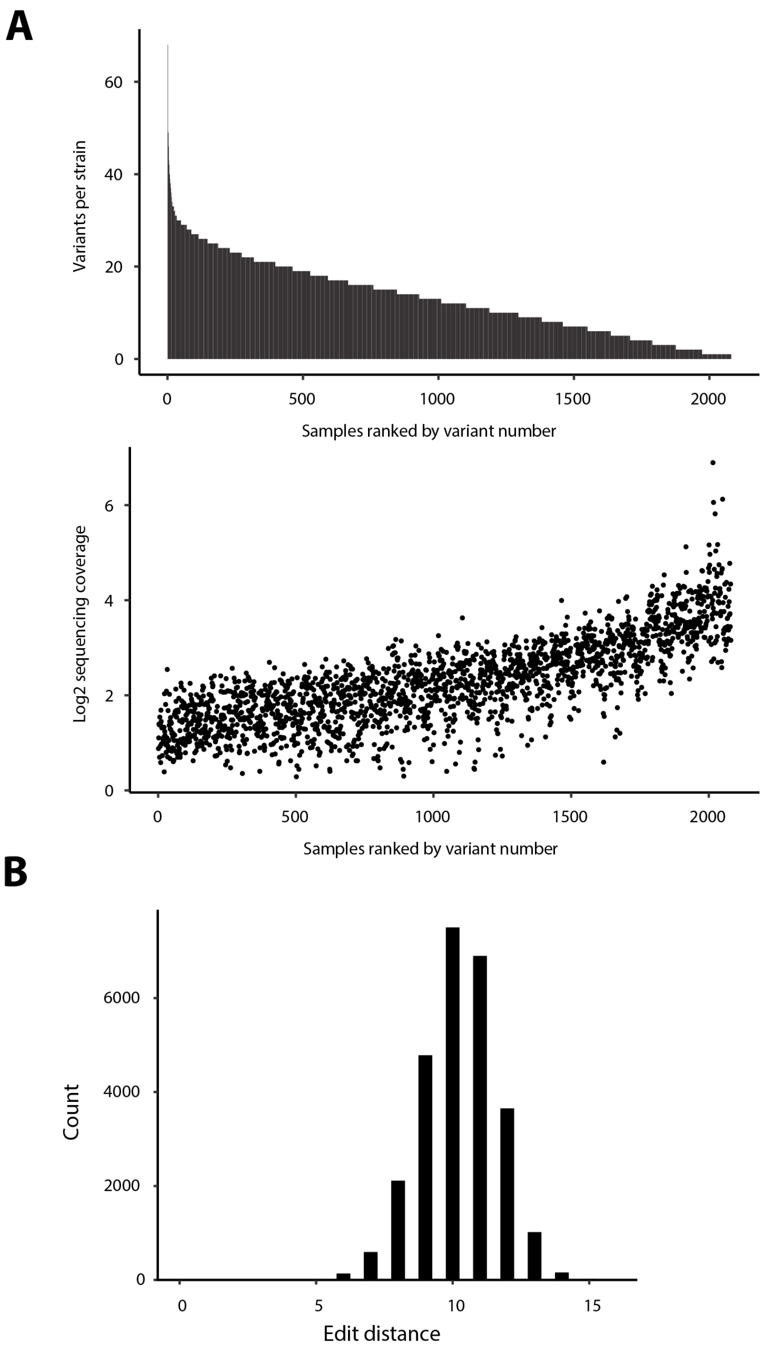

Figure S4. Survey of genomic variants across 2,260 genomes for off-target mutations

(**A**) The top panel shows the number of SNPs and indels found in each clone, arranged in order of variant count. The bottom panel displays the log2-transformed sequencing depth, arranged in the same order as the samples in the top panel. The correlation between higher variant number and lower sequencing depth indicates a higher false positive rate in this fraction of samples, and suggests the true rate of off-target or spontaneous mutations during editing and subsequent culturing steps is low. (**B**) The distribution of edit distance between the best match sequence within a 50 bp region around the variant, identified by sequence alignment, and the corresponding gRNA.

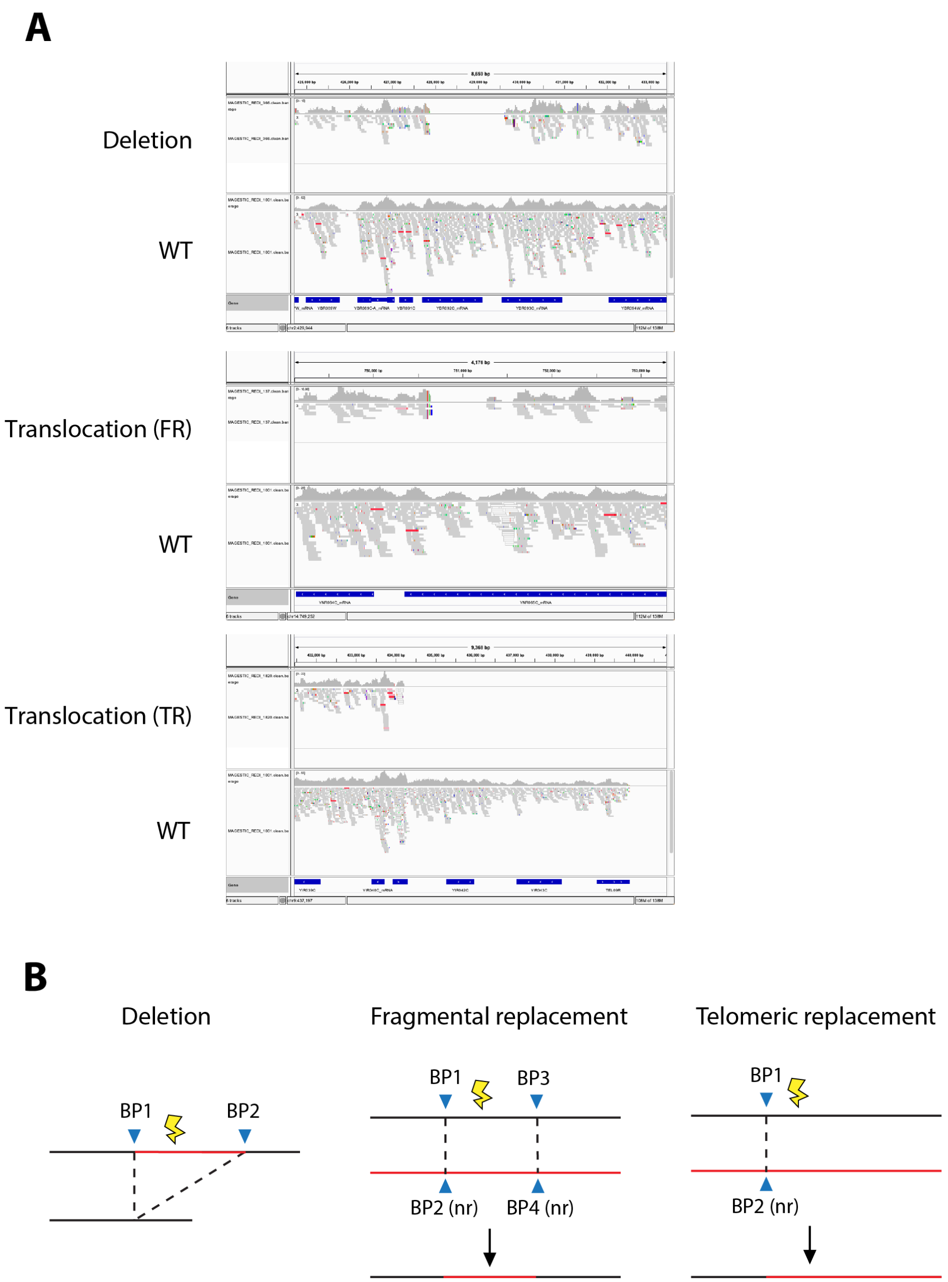

Figure S5. Visualization of SVs from short-read alignment data

(**A**) Snapshots in IGV for three clones at their SV regions. Mapping coverage was depleted at the target site for translocations due to their non-reciprocal nature. FR: fragmental replacement; TR: telomeric replacement; WT: wild-type. (**B**) SV breakpoints (BP) are defined as the first mismatched base/indel near the target site or the distal template site involved in DNA repair. A breakpoint marks the precise location where two distant genomic regions become abnormally joined. In fragmental and telomeric replacements, the breakpoint(s) on the template region do not undergo actual sequence rearrangement but instead serve solely as a repair template, resulting in a non-reciprocal (nr) structural variation. The cut site introduced by the nuclease is indicated in yellow.

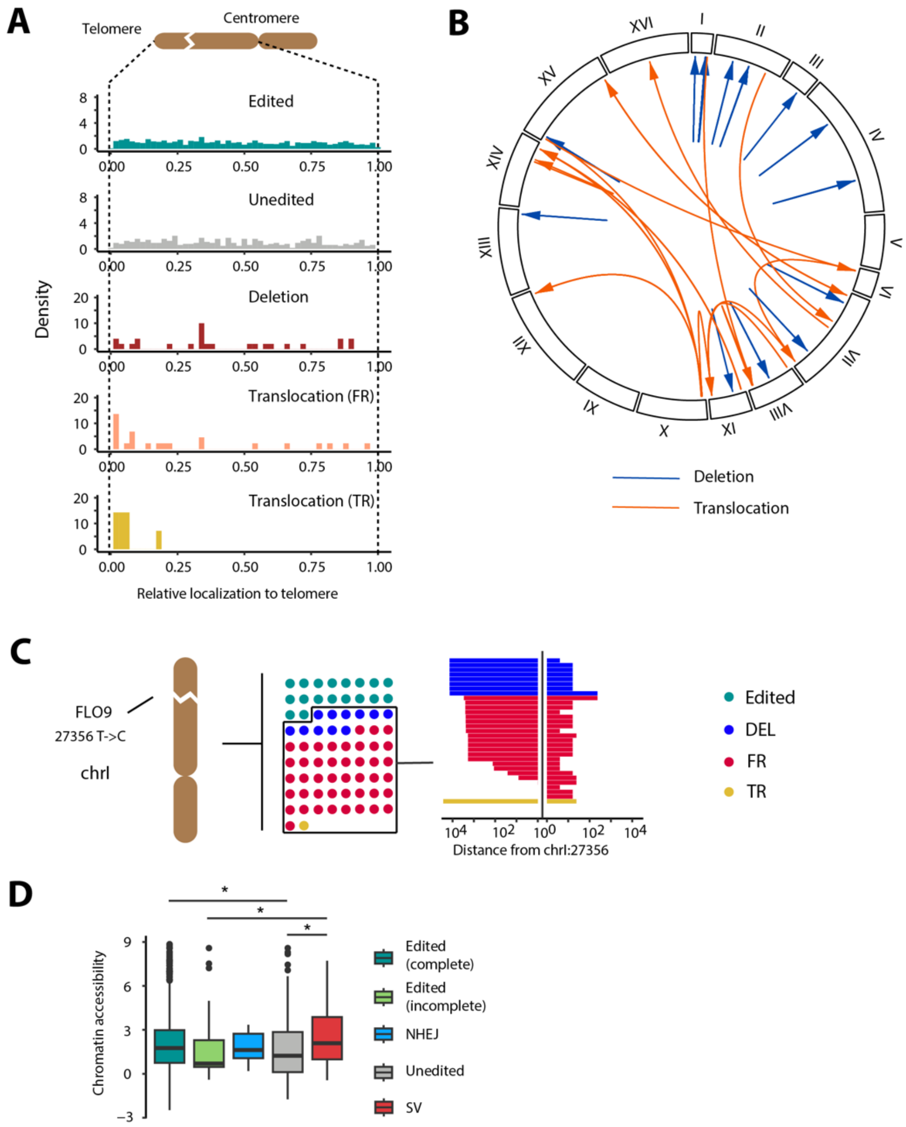

Figure S6. Cas9-induced SVs are dependent on genomic context

(**A**) Distribution of editing outcomes along the chromosome arm. The relative position of DSBs is shown with the telomeric end set as 0, and the centromeric end normalized to 1. FR: fragmental replacement; TR: telomeric replacement. (**B**) SV breakpoints were scanned to identify endogenous homologous regions used to template repair, with arrows indicating the direction of non-reciprocal translocations. The genomic sequences at the base of the arrows were used as template to repair the target region at arrow heads. (**C**) The exact start points (most telomeric SNV) and end points (most centromeric SNV) of SVs occurred when targeting SV_site_1 shown in **Fig. 1F**. Colored bars represent the window from the start to the end of the deleted fragment (DEL; blue) or replaced fragment (FR; red; or TR; yellow). Only clones with clear SV junction classified were included in the visualization. (**D**) Chromatin accessibilities are indicated by Tn5 insertion frequency derived from the public dataset. Data processing is detailed in **Supplementary Text**. Statistical significance was assessed by Kruskal-Wallis's one-way ANOVA test, followed by pairwise Mann-Whitney Wilcoxon's rank sum test, with p-values corrected by Benjamini-Hochberg's approach. *: p-value < 0.05.

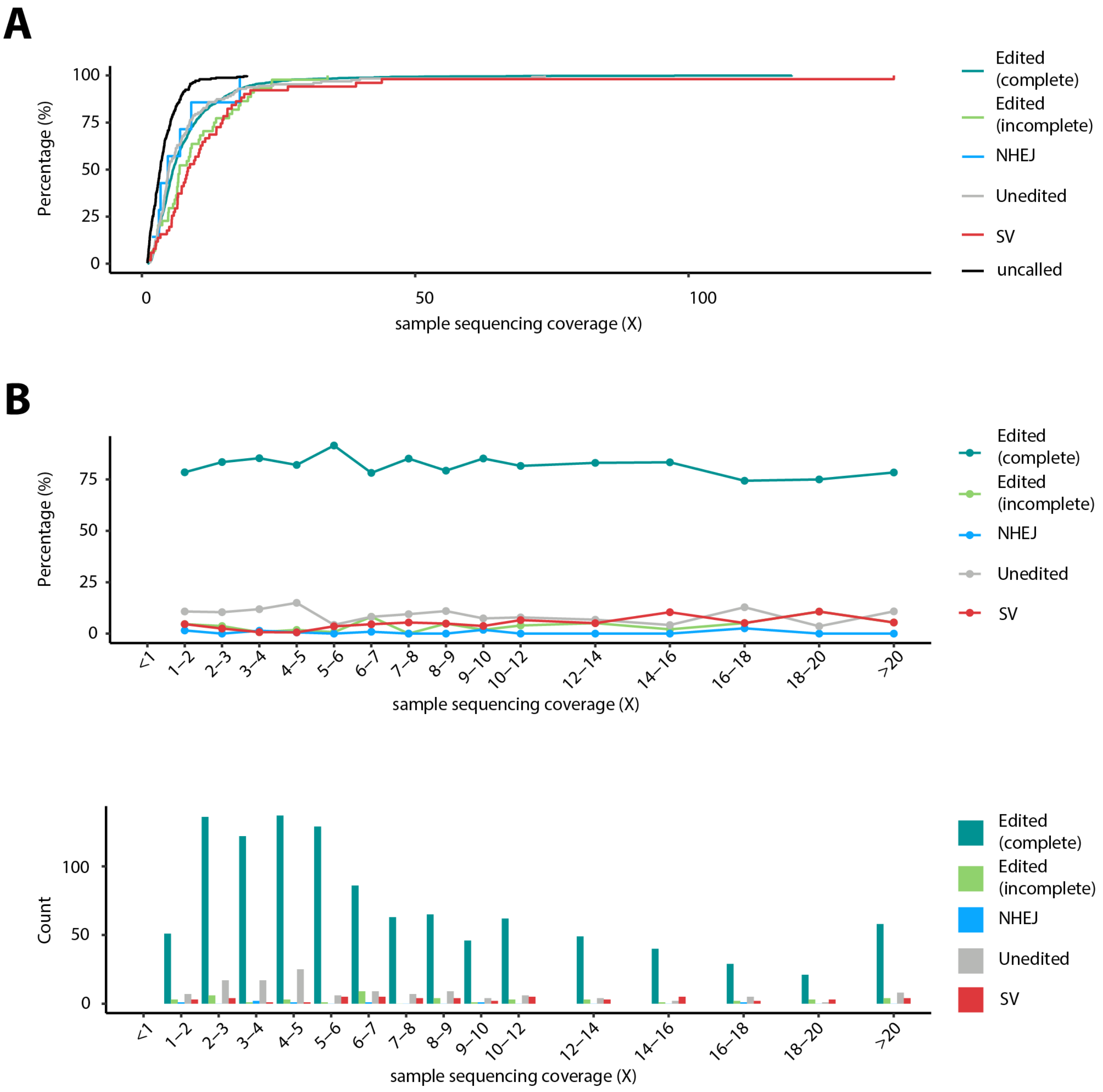

Figure S7. Detection bias of editing outcomes across different sequencing coverage.

(**A**) Cumulative distribution of detected editing outcome classes across samples with varying sequencing coverages. Only MAGESTIC v1.3 samples are included to ensure a consistent edit-to-unedit ratio. Detection rates are normalized to percentages (0-100%), representing the fraction of samples in which each outcome class was detected at or below the given sequencing coverage (X). (**B**) Distribution of detected editing outcome stratified by bins of sample sequencing coverage. The top panel shows the percentage distribution of outcome classes within each bin; the bottom panel shows the absolute number of samples in each class.

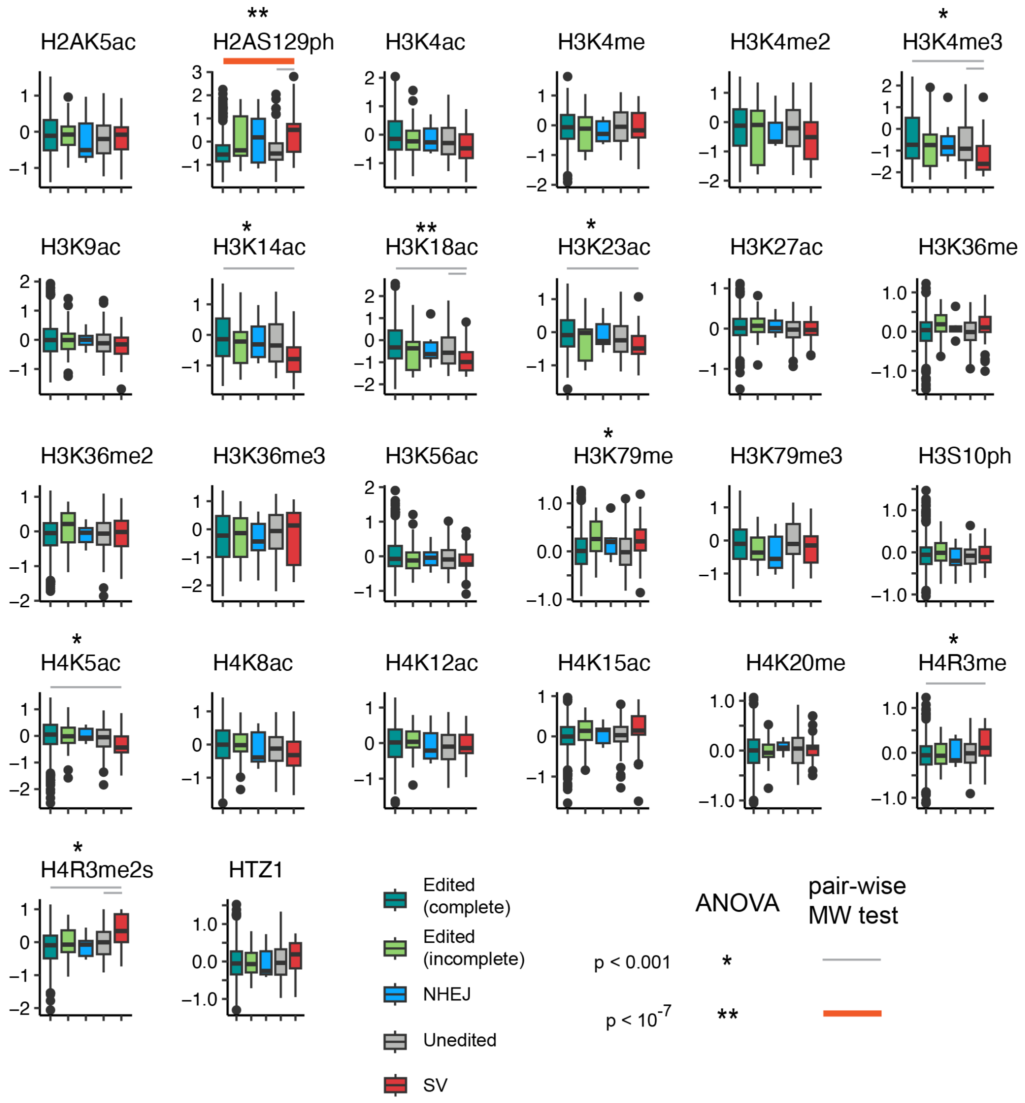

Figure S8. Histone modification profiles correlate with CRISPR/Cas9 editing outcome

Histone modification levels were quantified by the ratio of ChIP-seq signal intensity and input intensity at log scale (see **Supplementary Text**). Statistical significance was assessed by Kruskal-Wallis's one-way ANOVA test, followed by pairwise Mann-Whitney Wilcoxon's rank sum test, with p-values corrected by Benjamini-Hochberg's approach.

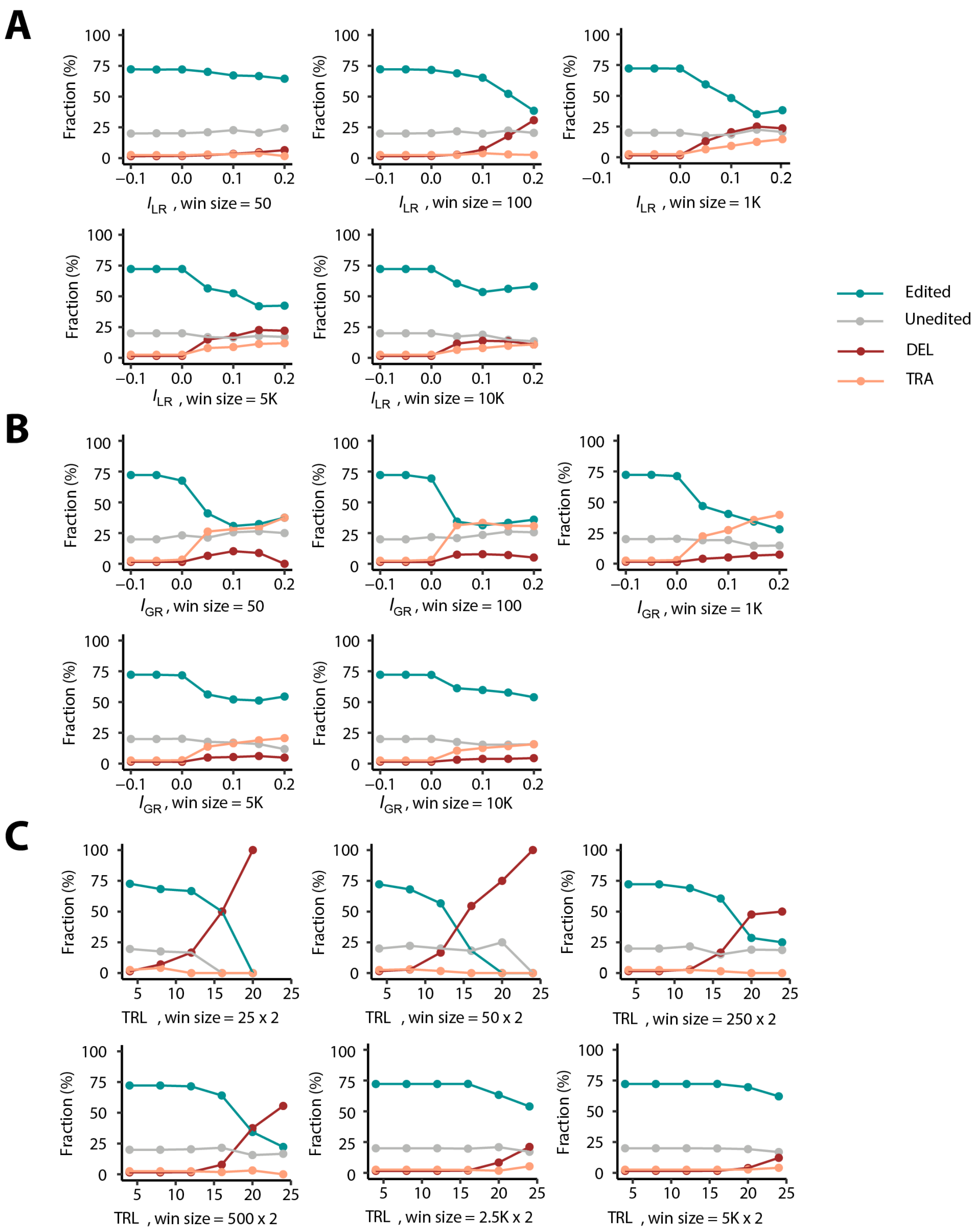

Figure S9. Repetitiveness indexes and tandem repeat length (TRL) across various window sizes

A gradient of window sizes was applied to define a region centered at the target PAM for calculating I_{LR}, I_{GR} (**A**, **B**) and TRL (**C**). For each window size, cutoffs were set for the minimum value of the corresponding metric to display the change in editing outcome proportions from least to most repetitive fractions of the genome. DEL: deletion; TRA: translocation.

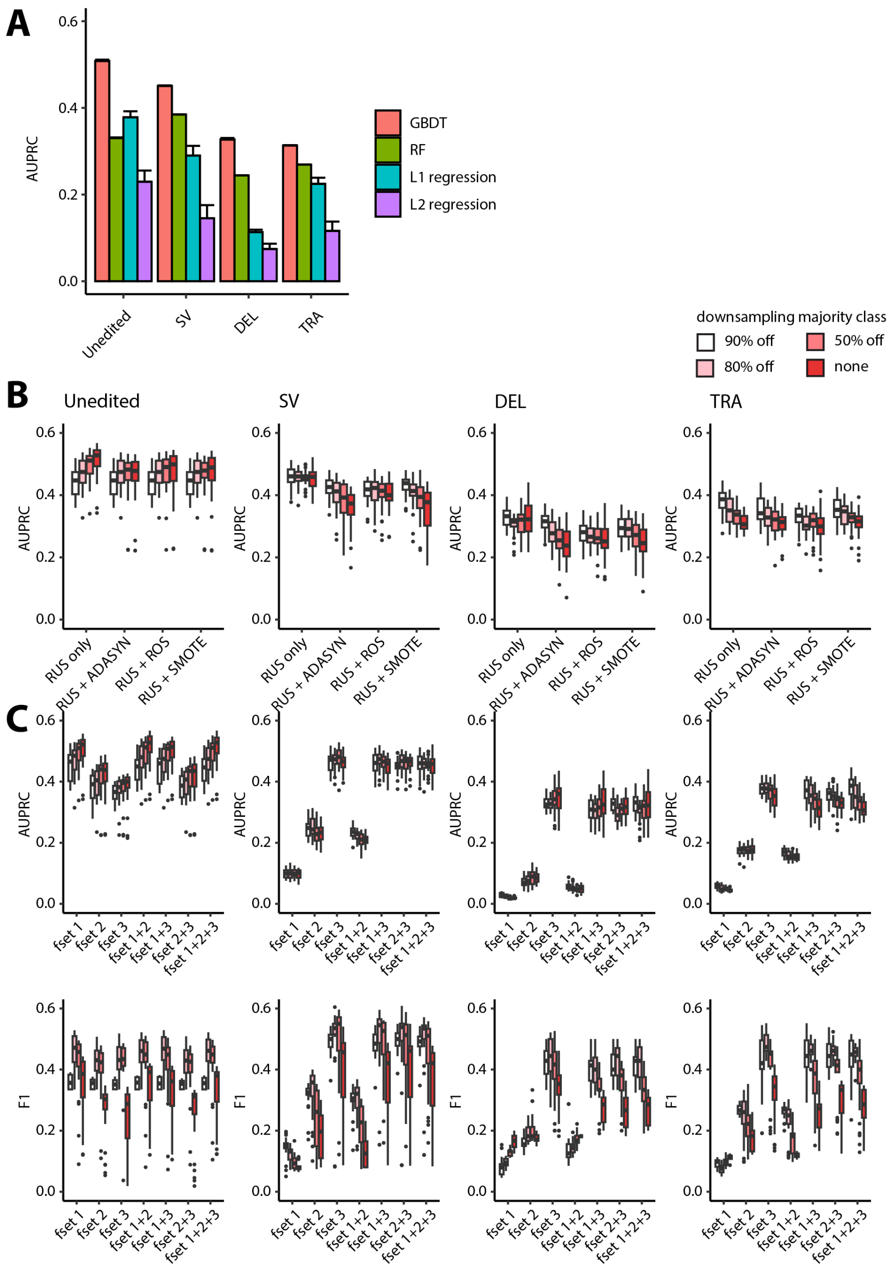

Figure S10. Comparison of model algorithms, downsampling strategies and feature sets
(**A**) Four machine-learning (ML) algorithms were compared for their performance on the training dataset using repeated k-fold cross validation (folds = 3, repeats = 5). Each model was trained with a grid search of corresponding hyperparameters. The AUPRC values across models resulting from each ML algorithm were grouped and shown as boxplots. (**B**) Downsampling of the majority class (correct edit) was performed at different cutoffs (90%, 80%, 50% or not applied), in combination with three different approaches to oversample the minority class. RUS: random under-sampling; ROS: random over-sampling; ADASYN: adaptive synthetic sampling; SMOTE: Synthetic minor over-sampling. (**C**) Three feature subsets were used alone or in combinations for model training and compared. The two technical factors (**table S5**) are involved in all models.

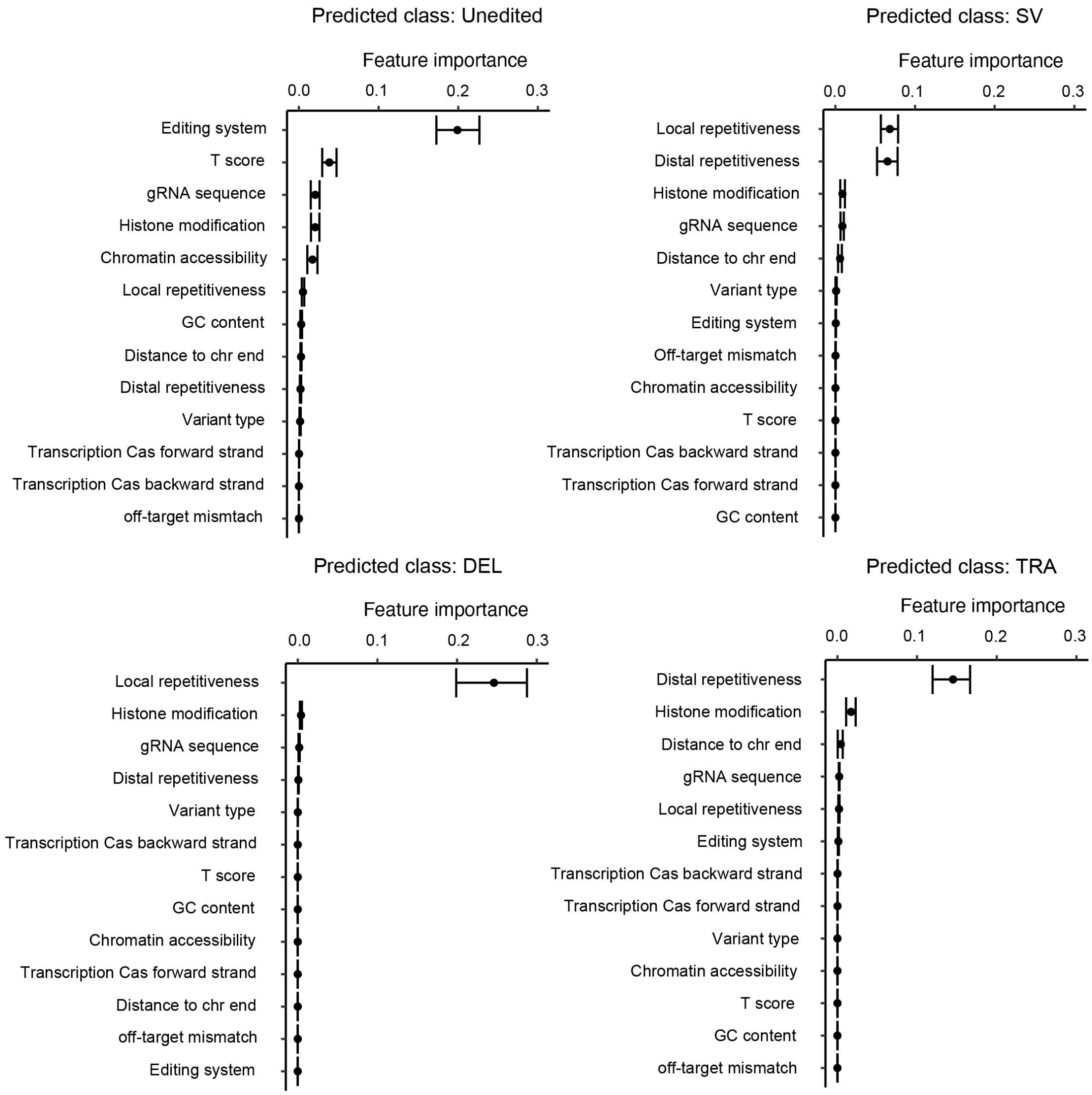

Figure S11. Analysis of feature importance for GBDT models

Feature importance calculated for the four gradient-boosted decision tree models trained for distinct outcome categories. Error bars denotes the 90% confidence intervals of ΔAUROC across 1,000 permutations.

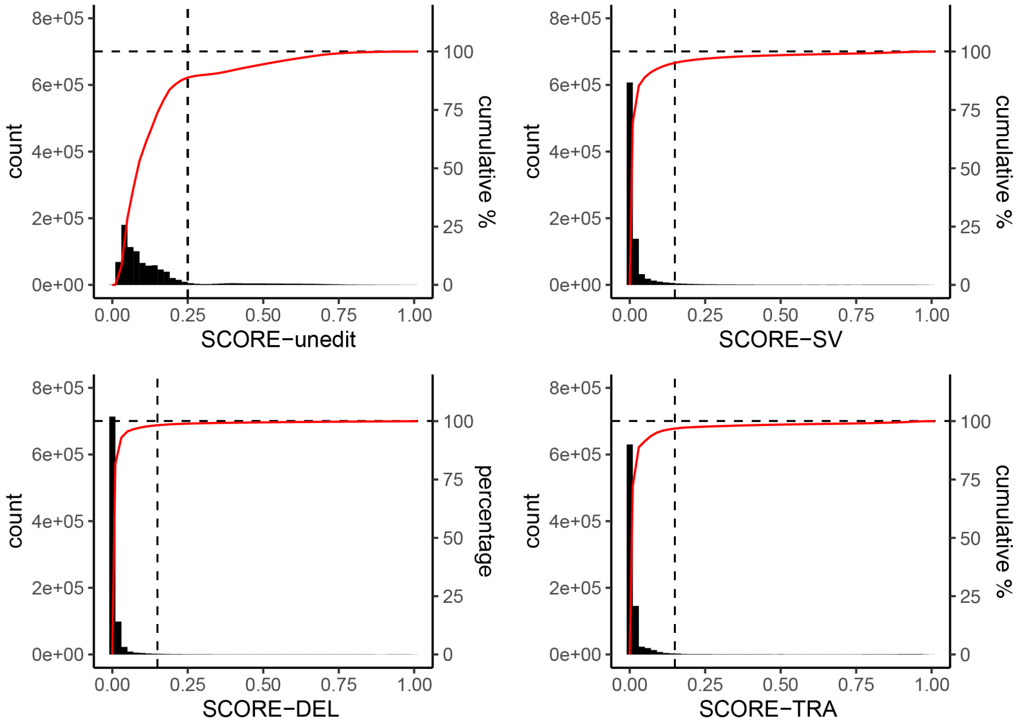

Figure S12. Empirical distributions of SCORE predictive values for 873,419 NGG sites

Scores for unedited, SV, DEL and TRA loci across 873,419 NGG positions on the yeast reference genome. The histograms display the density (counts) of the scores (use left vertical axis), with the red line indicating the cumulative distribution of values as percentage (use right vertical axis). The cutoffs for defining the tails of each distribution are indicated as vertical dashed lines. The horizontal dashed line denotes saturation of the cumulative distribution curve at 100%.

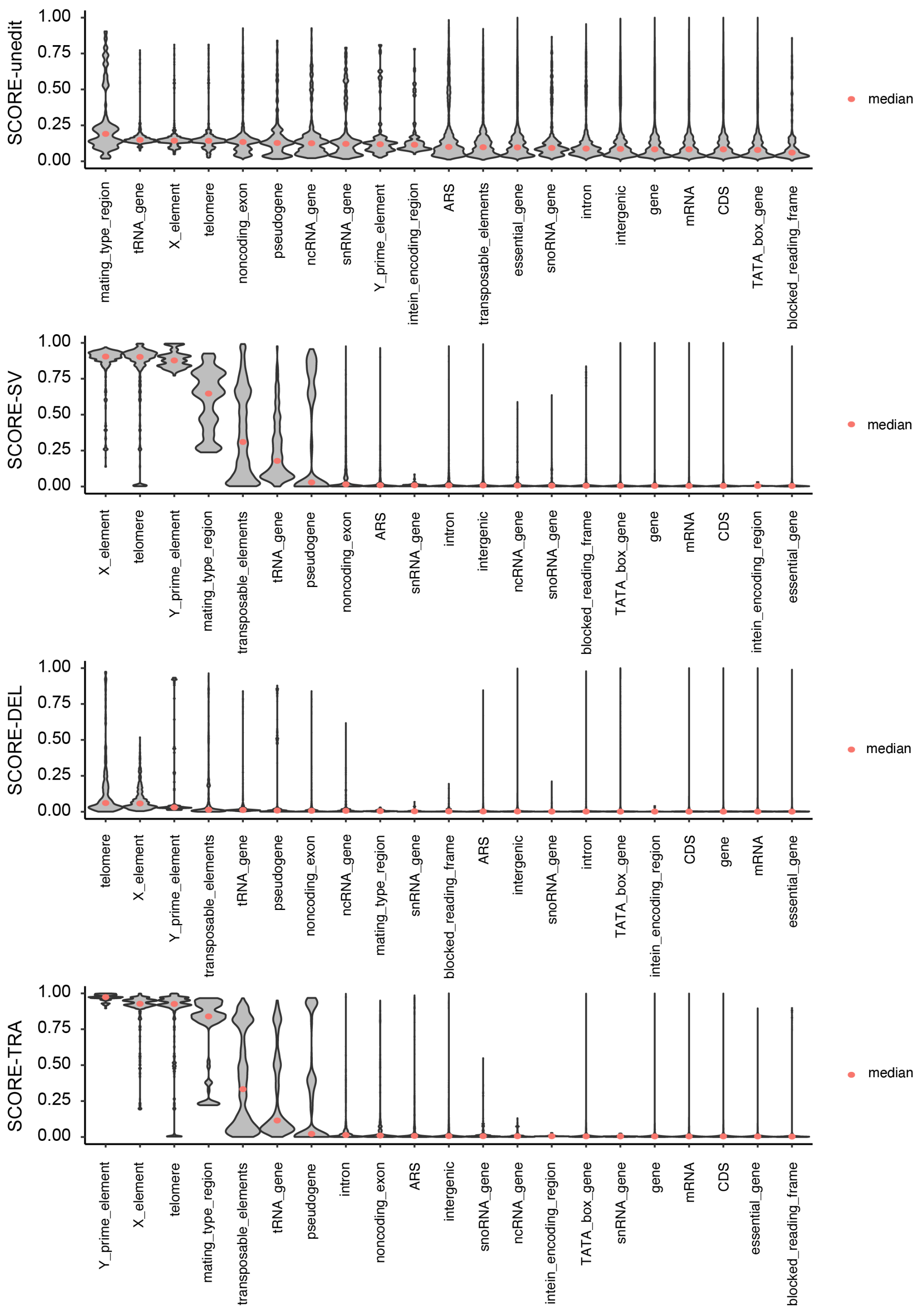

Figure S13. Predicted editing outcomes for genomic sites overlapping functional elements

Distribution of predicted probability of no editing, SV, DEL or TRA events across annotated functional elements throughout the yeast genome. Displayed elements were ordered by the median value of predicted scores.

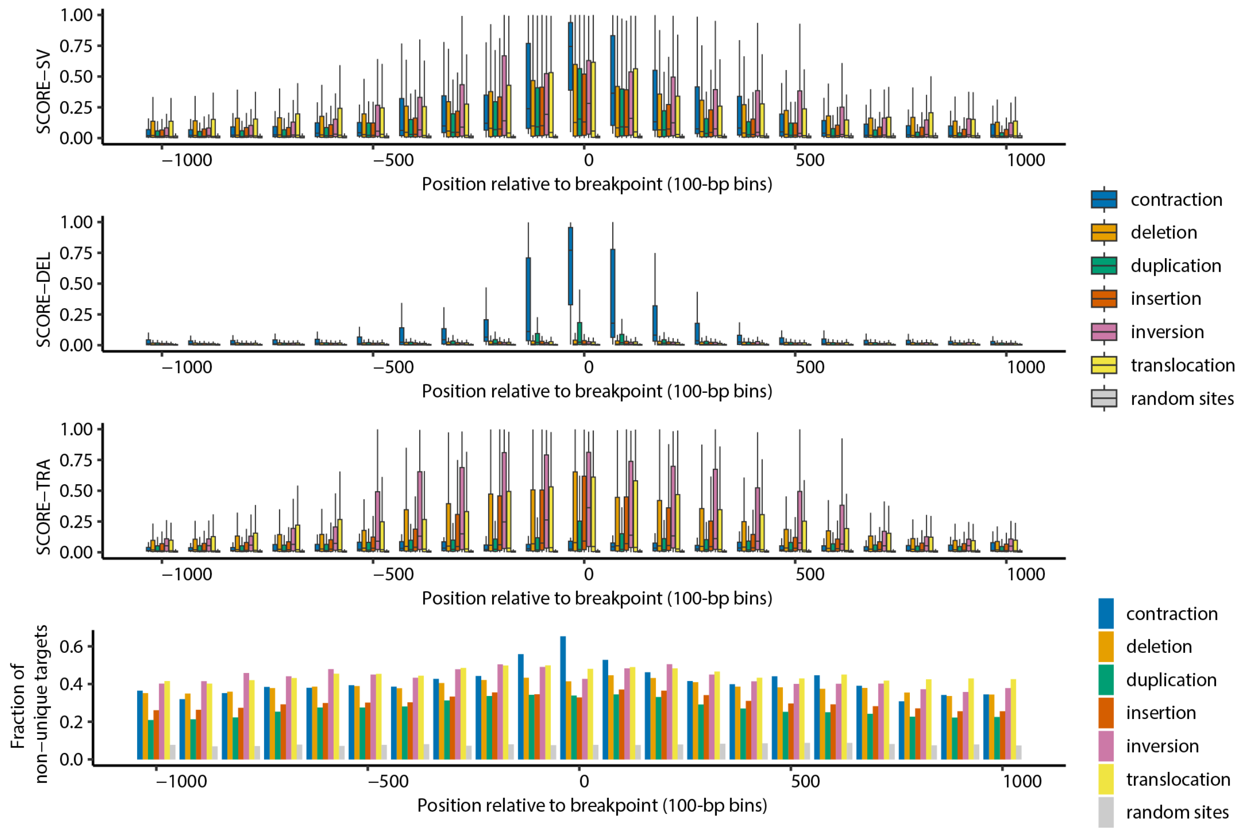

Figure S14. Predicted SV-prone genomic sites co-localize with natural SV breakpoints in *S. cerevisiae*

Distribution of predicted risk scores for SV, DEL and TRA formation at sites proximal to 9,618 natural SV breakpoints. Genomic positions were binned using 100-bp windows centered at each SV breakpoint (e.g., position 0 represents the window from -50 to +49 bp). Non-unique guide positions (i.e., guides that match more than a single genomic location) were pre-excluded from SCORE predictions, and are quantified separately as their fraction of total sites per bin (shown in the bottom panel). 1,000 random sites from the yeast genome, together with their up- and down-stream PAM sites are included as a control.

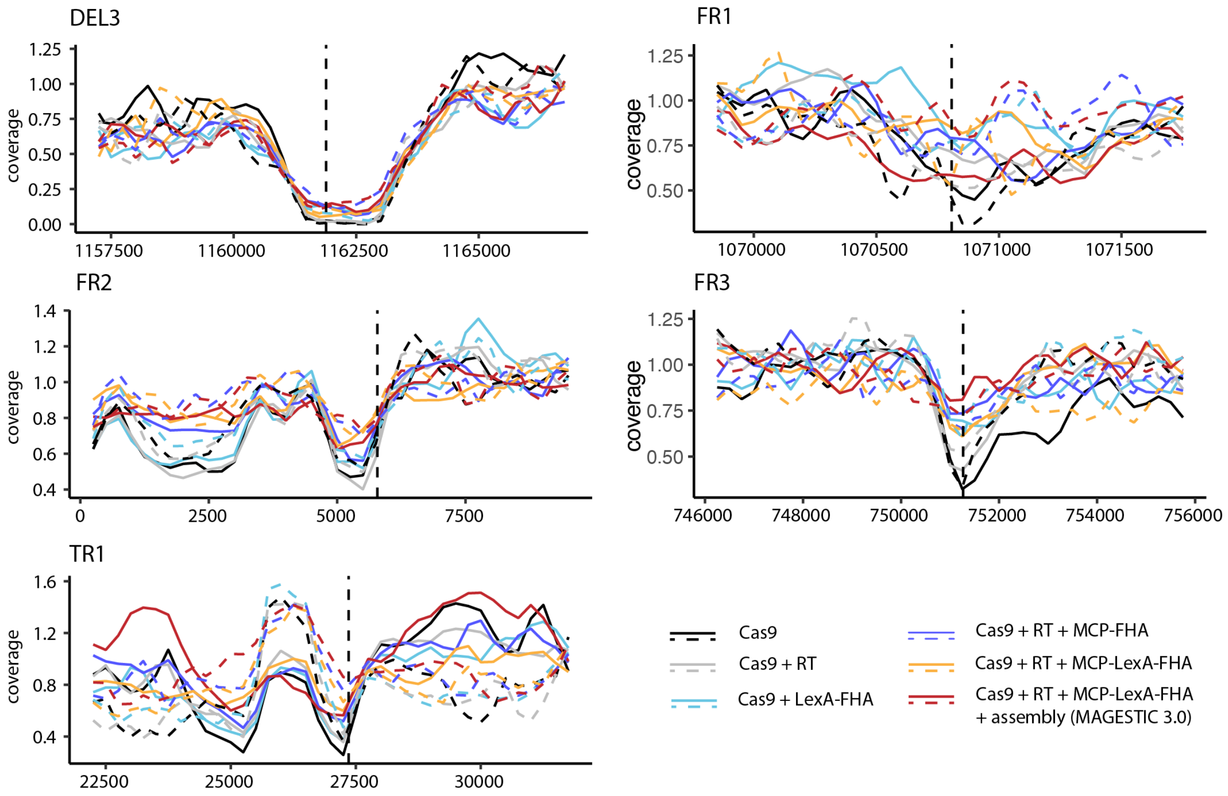

Figure S15. Comparison of HDR-enhancing editing systems targeting difficult-to-edit regions

A total of seven targets causing large deletions or translocations (five shown here and others in **Fig. 3B**) were investigated through bulk whole-genome sequencing, and the mapping coverage was used to detect the presence of SVs across six editing systems. A sliding window with a length of 1/25 the length of the displayed region and a step of 1/50 the length of the displayed region was used to compute the mapping coverage around the target PAM site (dashed line). The coverage value was normalized to control samples (unedited) representing the value of 1.0.

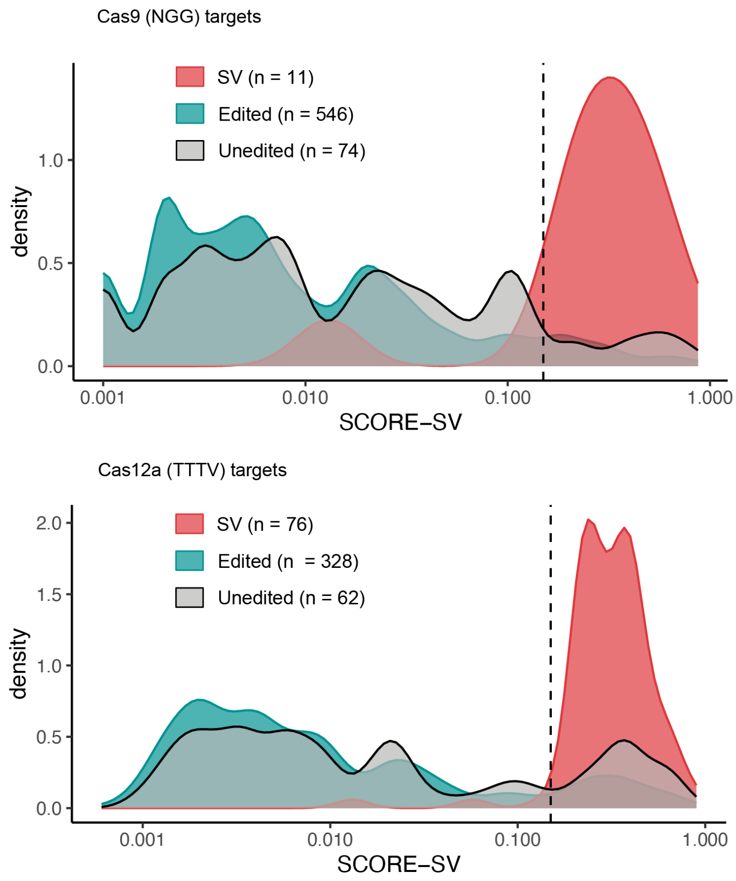

Figure S16. SCORE-SV prediction for independent CRISPR/Cas9 and CRISPR/Cas12a libraries.

WGS-confirmed editing outcomes of a gRNA-donor library targeting diverse genes located in previously mapped yeast QTLs. Targets were stratified by nuclease types: Cas9 (NGG PAM, upper panel), Cas12a (TTTV PAM, lower panel). The dashed line denotes the SCORE-SV threshold at 0.15.

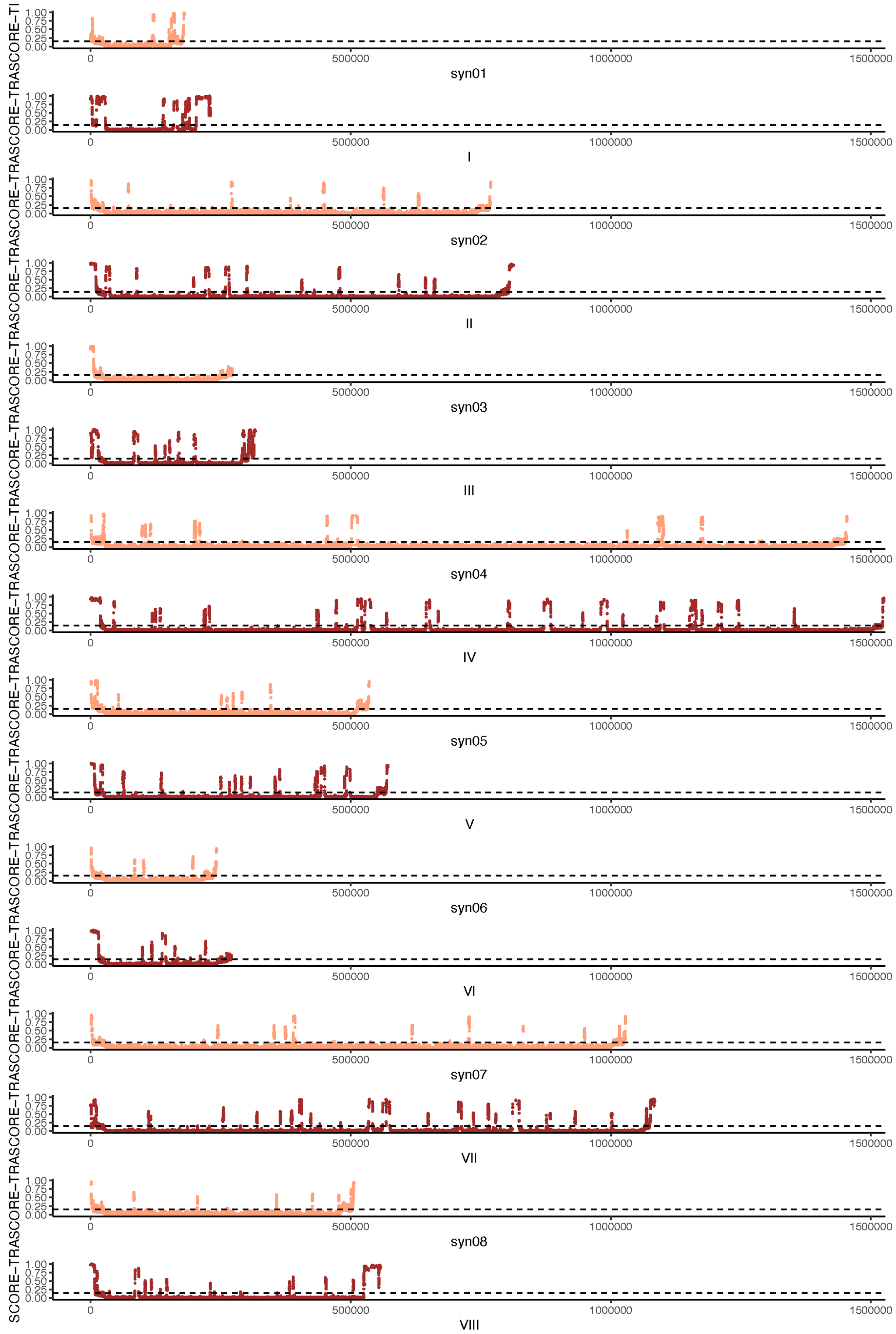

Figure S17. SCORE-TRA distribution in reference and synthetic yeast chromosomes (continuing on next page) (1/2)

SCORE-TRA prediction of translocation likelihood for all NGG sites in the two genomes (light salmon: Sc2.0 synthetic genome; brown: S288C reference genome).

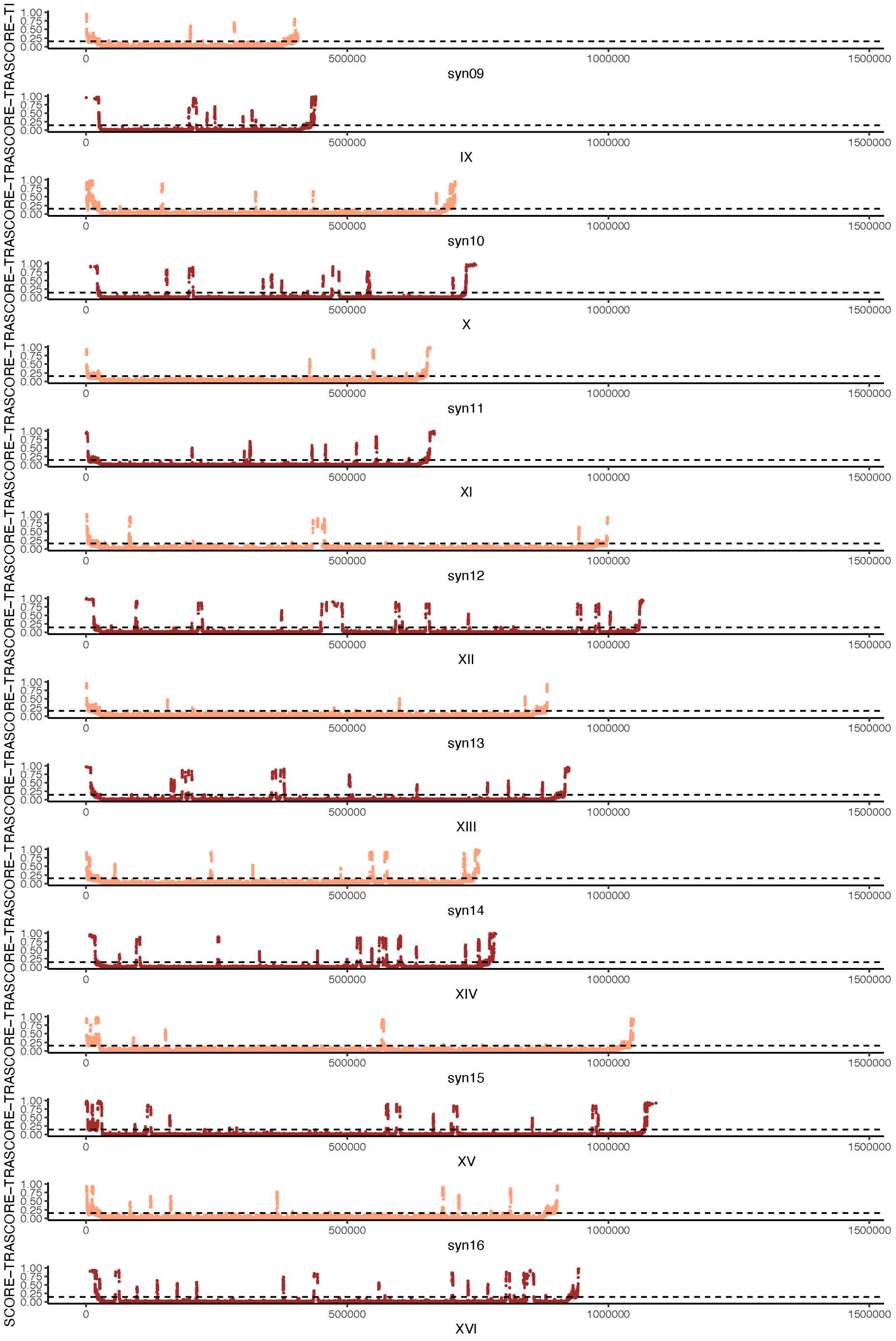

Figure S17. SCORE-TRA distribution in reference and synthetic yeast chromosomes (continuing from previous page) (2/2)

SCORE-TRA prediction of translocation likelihood for all NGG sites in the two genomes (light salmon: Sc2.0 synthetic genome; brown: S288C reference genome).

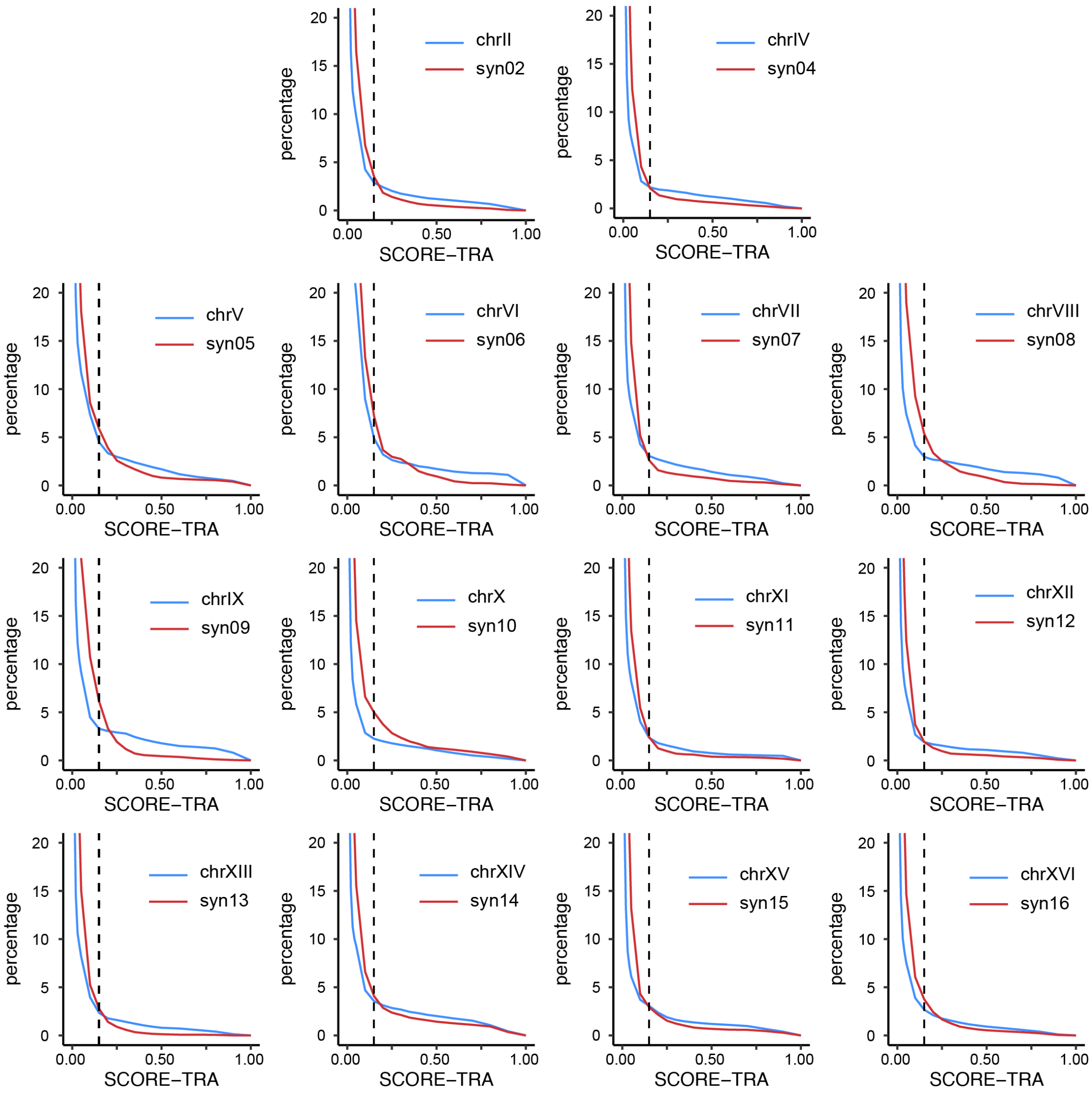

Figure S18. Distal sequence repetitiveness of reference and synthetic genomes

Cumulative distribution of SCORE-TRA in S288C (blue) vs. Sc2.0 (red) chromosomes. Results for chromosome 1 and 3 are shown in **Fig. 5B**.

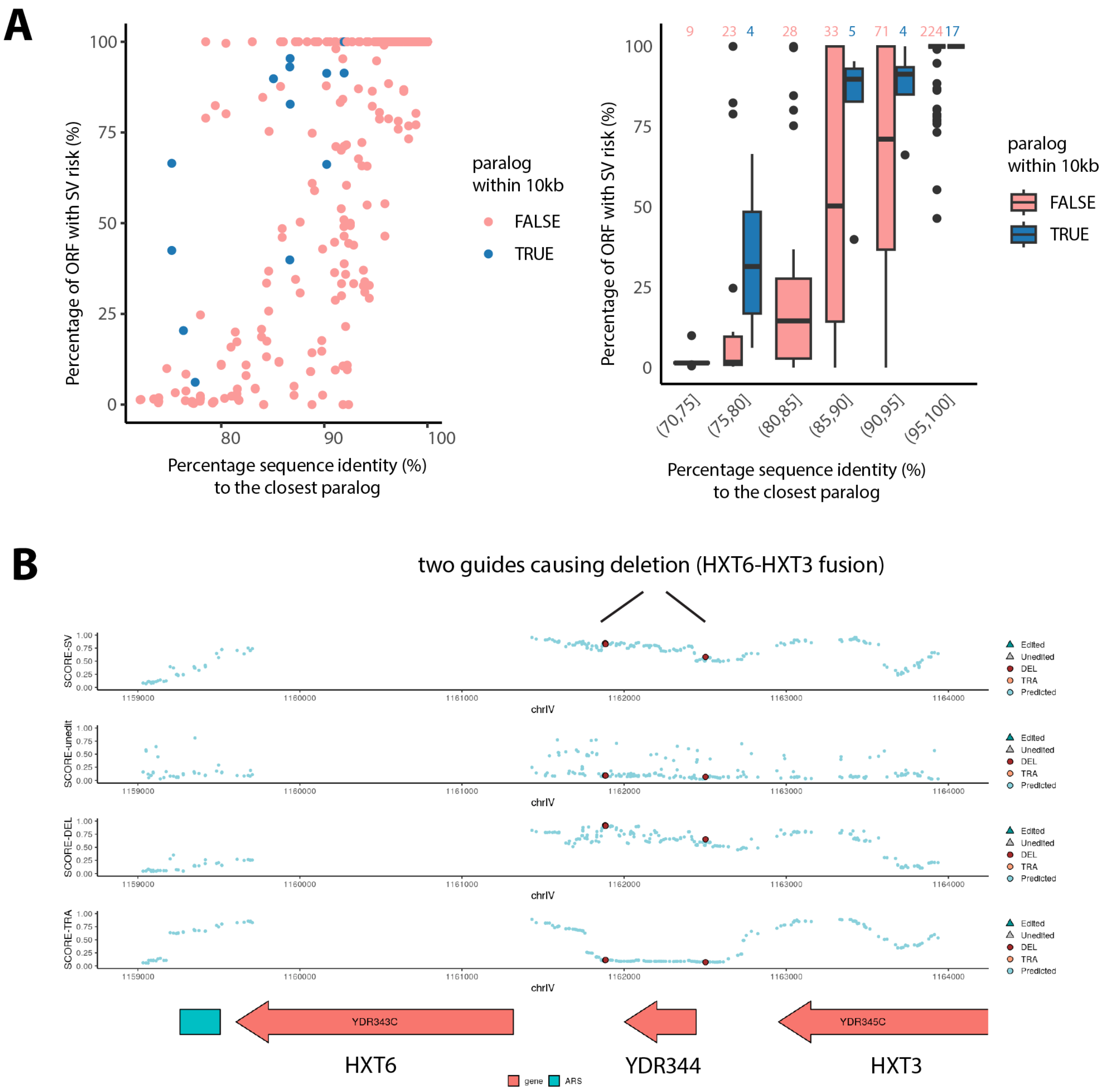

Figure S19. SV-prone sites arisen from homologous genes and their flanking contexts

(**A**) Homologous gene pairs were identified using a filtered list of BLASTN hits and classified as proximal or distal pairs based on a 10kb distance threshold. An SV-risk site is defined as an NGG site with a SCORE-SV > 0.15 or one that contains an off-target match elsewhere in the genome. The SV-risk level (i.e. y-axis) for each ORF is calculated as the number of SV-risk sites divided by the total number of NGG sites within that ORF. Box plot (right) shows the distribution of ORF SV-risk levels stratified by bins of sequence identity to the closest paralog. The number of genes represented in each box plot is shown above the corresponding box. (**B**) Screenshot of the region browser was taken from the SCORE website focusing on the *HXT6-HXT3* locus. Two deletion events were highlighted as dark red dots indicating samples confirmed in the 1,875 WGS dataset. This is an example where Cas9 targeting non-repetitive sequences (UTRs of YDR344) leads to SVs due to their proximity to repetitive sequences (*HXT6, HXT3*).

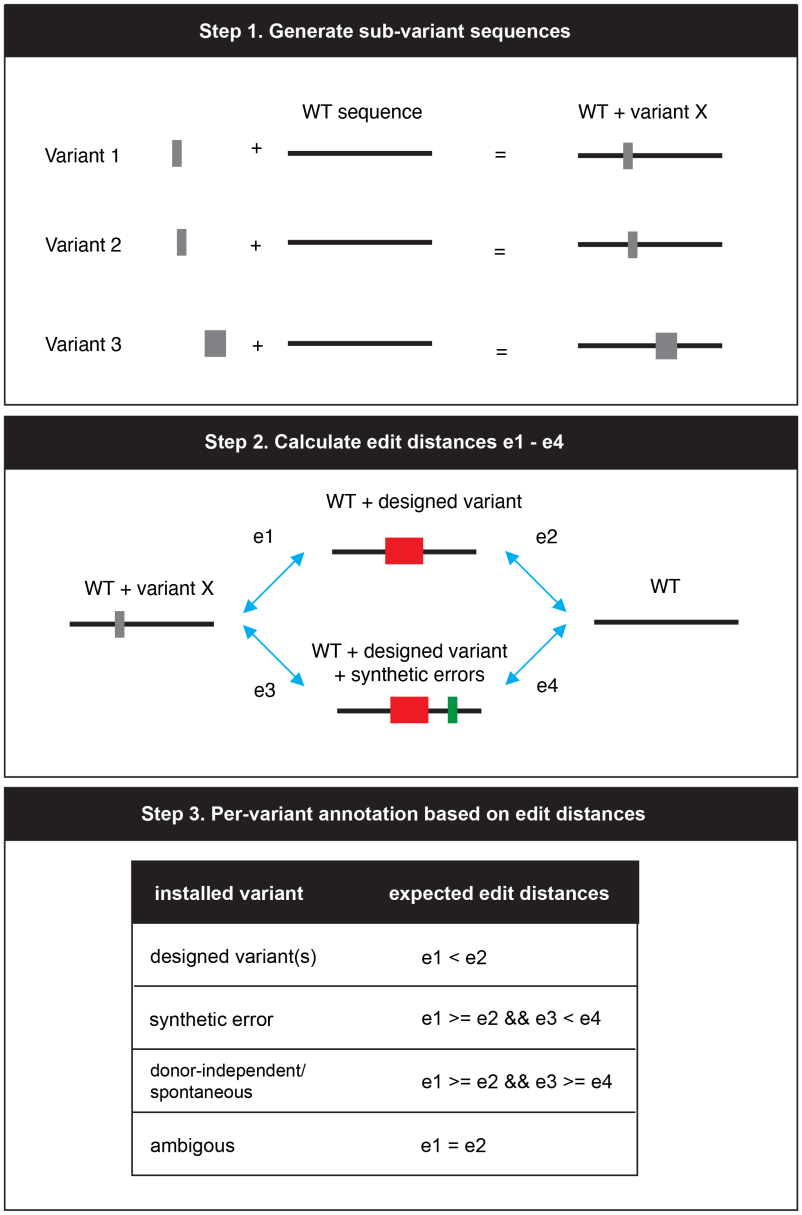

Figure S20. Scheme for classification of variants identified by whole-genome sequencing
Classification of variants detected in WGS samples using GATK4 based on edit distances (e1 to e4) computed between observed sequence, wild-type sequence and donor template (with and without synthesis errors).

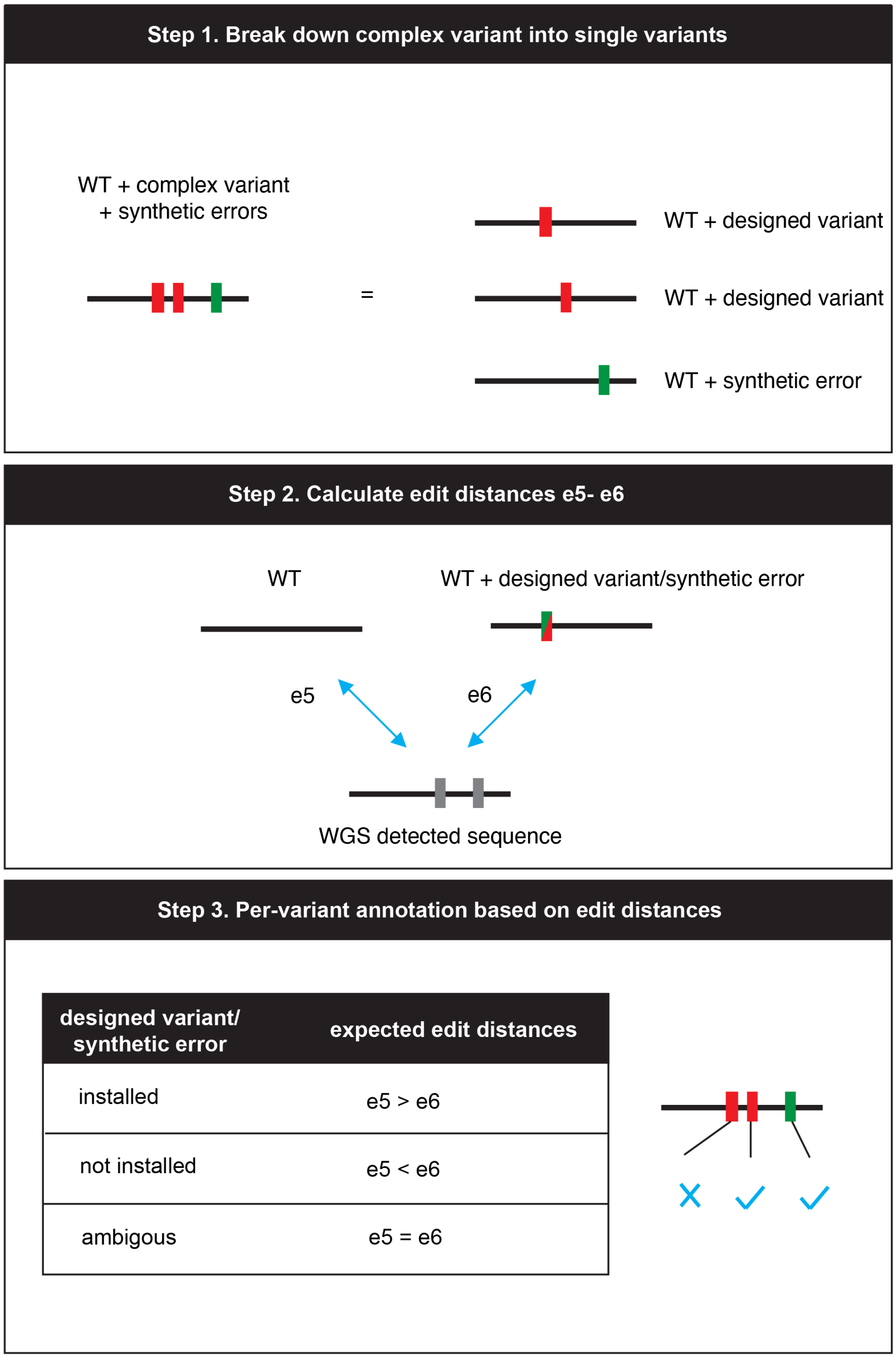

Figure S21. Scheme for classification of sub-variant incorporation status
Classification of sub-variant (of an MNV) incorporation status based on edit distances e5 and e6. The information obtained was used to define the co-installation of adjacent sub-variants on the donor template.

Supplementary Tables

Table S1. Summary of editing outcomes at 2,260 targeted sites

Clone, target variant, editing system and sequencing information for all the isolated clones in the training dataset.

Table S2. Annotation of homologous sequence pairs at SV breakpoints

Information of sequence homology found with the paired breakpoints in deletions and translocations.

Table S3. Summary of editing outcomes at 654 potential off-target sites with ≤3 mismatches.

Genome sites with ≤3 mismatches in the gRNA alignment flanked by an NGG motif extracted and assessed for off-target SV formation.

Table S4. Whole-genome sequencing of 404 clones edited at four target SV-prone sites.

Four sites with SCORE-SV > 0.15 were re-assessed for editing outcomes via re-cloning, editing and isolating single colonies for sequencing.

Table S5. Feature list used in prediction models

Features were grouped into three subsets for testing and training SCORE. Two additional correction factors are included in all models to exclude confounding effects.

Table S6. Parameters for the final SCORE models

List of final feature sets and training parameters for gradient-boosted decision trees used for training SCORE.

Table S7. Genomic fraction of Cas9 NGG motifs subject to unintended outcomes

The S288C genome is subdivided into regions of different levels of unintended outcome risks.

Table S8. SV hotspots across the yeast genome

Information of 562 identified SV hotspot regions in the S288C genome.

Table S9. Loci receiving structural variants assessed with bulk whole-genome sequencing.

Variant, gRNA and donor DNA information for SV-prone sites tested for variations of MAGESTIC systems.

Table S10. Strains, constructs and plasmids used in this study

Detailed information for strains, constructs and plasmids.

Table S11. Editing outcome of 1,122 independent clones assessed by whole-genome sequencing

Clone, target variant and sequencing information for all the isolated clones in the validation dataset.

Table S12. Summary of median- and high-TRA risk regions in S288C and Sc2.0 genomes

Fraction of chromosomes under risk of forming translocations, compared between the original S288C reference and the Sc2.0 synthetic genomes.

REFERENCES AND NOTES

1. R. E. Hanna, M. Hegde, C. R. Fagre, P. C. DeWeirdt, A. K. Sangree, Z. Szegletes, A. Griffith, M. N. Feeley, K. R. Sanson, Y. Baidi, L. W. Koblan, D. R. Liu, J. T. Neal, J. G. Doench, Massively parallel assessment of human variants with base editor screens. *Cell* **184**, 1064–1080.e20 (2021).

2. J. M. T. Hunt, C. A. Samson, A. du Rand, H. M. Sheppard, Unintended CRISPR-Cas9 editing outcomes: A review of the detection and prevalence of structural variants generated by gene-editing in human cells. *Hum. Genet.* **142**, 705–720 (2023).

3. A. Movahedi, S. Aghaei-Dargiri, H. Li, Q. Zhuge, W. Sun, CRISPR variants for gene editing in plants: Biosafety risks and future directions. *Int. J. Mol. Sci.* **24**, 16241 (2023).

4. C. Xue, E. C. Greene, DNA repair pathway choices in CRISPR-Cas9-mediated genome editing. *Trends Genet.* **37**, 639–656 (2021).

5. S. Papathanasiou, S. Markoulaki, L. J. Blaine, M. L. Leibowitz, C.-Z. Zhang, R. Jaenisch, D. Pellman, Whole chromosome loss and genomic instability in mouse embryos after CRISPR-Cas9 genome editing. *Nat. Commun.* **12**, 5855 (2021).

6. C. A. Tsuchida, N. Brandes, R. Bueno, M. Trinidad, T. Mazumder, B. Yu, B. Hwang, C. Chang, J. Liu, Y. Sun, C. R. Hopkins, K. R. Parker, Y. Qi, L. Hofman, A. T. Satpathy, E. A. Stadtmauer, J. H. D. Cate, J. Eyquem, J. A. Fraietta, C. H. June, H. Y. Chang, C. J. Ye, J. A. Doudna, Mitigation of chromosome loss in clinical CRISPR-Cas9-engineered T cells. *Cell* **186**, 4567–4582.e20 (2023).

7. M. L. Leibowitz, S. Papathanasiou, P. A. Doerfler, L. J. Blaine, L. Sun, Y. Yao, C.-Z. Zhang, M. J. Weiss, D. Pellman, Chromothripsis as an on-target consequence of CRISPR–Cas9 genome editing. *Nat. Genet.* **53**, 895–905 (2021).

8. M. Fiumara, *S. Ferrari*, A. Omer-Javed, S. Beretta, L. Albano, D. Canarutto, A. Varesi, C. Gaddoni, C. Brombin, F. Cugnata, E. Zonari, M. M. Naldini, M. Barcella, B. Gentner, I. Merelli, L. Naldini, Genotoxic effects of base and prime editing in human hematopoietic stem cells. *Nat. Biotechnol.* **42**, 877–891 (2024).

9. G.-H. Hwang, S.-H. Lee, M. Oh, S. Kim, O. Habib, H.-K. Jang, H. S. Kim, Y. Kim, C. H. Kim, S. Kim, S. Bae, Large DNA deletions occur during DNA repair at 20-fold lower frequency for base editors and prime editors than for Cas9 nucleases. *Nat. Biomed. Eng.* **9**, 79–92 (2024).

10. L. Wu, S. Jiang, M. Shi, T. Yuan, Y. Li, P. Huang, Y. Li, E. Zuo, C. Zhou, Y. Sun, Adenine base editors induce off-target structure variations in mouse embryos and primary human T cells. *Genome Biol.* **25**, 291 (2024).

11. M. E. Huang, Y. Qin, Y. Shang, Q. Hao, C. Zhan, C. Lian, S. Luo, L. D. Liu, S. Zhang, Y. Zhang, Y. Wo, N. Li, S. Wu, *T. Gui*, B. Wang, Y. Luo, Y. Cai, X. Liu, Z. Xu, P. Dai, S. Li, L. Zhang, J. Dong, J. Wang, X. Zheng, Y. Xu, Y. Sun, W. Wu, L.-S. Yeap, F.-L. Meng, C-to-G editing generates double-strand breaks causing deletion, transversion and translocation. *Nat. Cell Biol.* **26**, 294–304 (2024).

12. J. G. Doench, N. Fusi, M. Sullender, M. Hegde, E. W. Vaimberg, K. F. Donovan, I. Smith, Z. Tothova, C. Wilen, R. Orchard, H. W. Virgin, J. Listgarten, D. E. Root, Optimized sgRNA design to maximize activity and minimize off-target effects of CRISPR-Cas9. *Nat. Biotechnol.* **34**, 184–191 (2016).

13. N. Kim, H. K. Kim, S. Lee, J. H. Seo, J. W. Choi, J. Park, S. Min, S. Yoon, S.-R. Cho, H. H. Kim, Prediction of the sequence-specific cleavage activity of Cas9 variants. *Nat. Biotechnol.* **38**, 1328–1336 (2020).

14. M. W. Shen, M. Arbab, J. Y. Hsu, D. Worstell, S. J. Culbertson, O. Krabbe, C. A. Cassa, D. R. Liu, D. K. Gifford, R. I. Sherwood, Predictable and precise template-free CRISPR editing of pathogenic variants. *Nature* **563**, 646–651 (2018).

15. G. Martínez-Gálvez, P. Joshi, I. Friedberg, A. Manduca, S. C. Ekker, Deploying MMEJ using MENdel in precision gene editing applications for gene therapy and functional genomics. *Nucleic Acids Res.* **49**, 67–78 (2020).

16. K. R. Roy, J. D. Smith, S. C. Vonesch, G. Lin, C. S. Tu, A. R. Lederer, A. Chu, S. Suresh, M. Nguyen, J. Horecka, A. Tripathi, W. T. Burnett, M. A. Morgan, J. Schulz, K. M. Orsley, W. Wei,

R. S. Aiyar, R. W. Davis, V. A. Bankaitis, J. E. Haber, M. L. Salit, R. P. St Onge, L. M. Steinmetz, Multiplexed precision genome editing with trackable genomic barcodes in yeast. *Nat. Biotechnol.* **36**, 512–520 (2018).

17. J. D. Smith, U. Schlecht, W. Xu, S. Suresh, J. Horecka, M. J. Proctor, R. S. Aiyar, R. A. O. Bennett, A. Chu, Y. F. Li, K. Roy, R. W. Davis, L. M. Steinmetz, R. W. Hyman, S. F. Levy, R. P. St. Onge, A method for high-throughput production of sequence-verified DNA libraries and strain collections. *Mol. Syst. Biol.* **13**, 913 (2017).

18. S. C. Vonesch, S. Li, C. Szu Tu, B. P. Hennig, N. Dobrev, L. M. Steinmetz, Fast and inexpensive whole-genome sequencing library preparation from intact yeast cells. *G3 GenesGenomesGenetics* **11**, jkaa009 (2021).

19. C. H. Emerson, A. A. Bertuch, Consider the workhorse: Nonhomologous end-joining in budding yeast. *Biochem. Cell Biol.* **94**, 396–406 (2016).

20. J. E. DiCarlo, J. E. Norville, P. Mali, X. Rios, J. Aach, G. M. Church, Genome engineering in *Saccharomyces cerevisiae* using CRISPR-Cas systems. *Nucleic Acids Res.* **41**, 4336–4343 (2013).

21. G. I. Lang, A. W. Murray, Estimating the per-base-pair mutation rate in the yeast *Saccharomyces cerevisiae*. *Genetics* **178**, 67–82 (2008).

22. J. A. Downs, S. Allard, O. Jobin-Robitaille, A. Javaheri, A. Auger, N. Bouchard, S. J. Kron, S. P. Jackson, J. Côté, Binding of chromatin-modifying activities to phosphorylated histone H2A at DNA damage sites. *Mol. Cell* **16**, 979–990 (2004).

23. J. Seo, S. C. Kim, H.-S. Lee, J. K. Kim, H. J. Shon, N. L. M. Salleh, K. V. Desai, J. H. Lee, E.-S. Kang, J. S. Kim, J. K. Choi, Genome-wide profiles of H2AX and γ-H2AX differentiate endogenous and exogenous DNA damage hotspots in human cells. *Nucleic Acids Res.* **40**, 5965–5974 (2012).

24. D. M. Baird, Telomeres and genomic evolution. *Philos. Trans. R. Soc. B Biol. Sci.* **373**, 20160437 (2018).

25. D. D. G. Owens, A. Caulder, V. Frontera, J. R. Harman, A. J. Allan, A. Bucakci, L. Greder, G. F. Codner, P. Hublitz, P. J. McHugh, L. Teboul, M. F. T. R. de Bruijn, Microhomologies are prevalent at Cas9-induced larger deletions. *Nucleic Acids Res.* **47**, 7402–7417 (2019).

26. G. Cullot, E. J. Aird, M. F. Schlapansky, C. D. Yeh, L. van de Venn, I. Vykhlyantseva, S. Kreutzer, D. Mailänder, B. Lewków, J. Klermund, C. Montellese, M. Biserni, F. Aeschimann, C. Vonarburg, H. Gehart, T. Cathomen, J. E. Corn, Genome editing with the HDR-enhancing DNA-PKcs inhibitor AZD7648 causes large-scale genomic alterations. *Nat. Biotechnol.*, 10.1038/s41587-024-02488-6 (2024).

27. B. Haubold, T. Wiehe, How repetitive are genomes? *BMC Bioinformatics* **7**, 541 (2006).

28. X. Wu, D. A. Scott, A. J. Kriz, A. C. Chiu, P. D. Hsu, D. B. Dadon, A. W. Cheng, A. E. Trevino, S. Konermann, S. Chen, R. Jaenisch, F. Zhang, P. A. Sharp, Genome-wide binding of the CRISPR endonuclease Cas9 in mammalian cells. *Nat. Biotechnol.* **32**, 670–676 (2014).

29. A. D. Basehoar, S. J. Zanton, B. F. Pugh, Identification and distinct regulation of yeast TATA box-containing genes. *Cell* **116**, 699–709 (2004).

30. T. Kobayashi, Regulation of ribosomal RNA gene copy number and its role in modulating genome integrity and evolutionary adaptability in yeast. *Cell. Mol. Life Sci. CMLS* **68**, 1395–1403 (2011).

31. S. O'Donnell, J.-X. Yue, O. A. Saada, N. Agier, C. Caradec, T. Cokelaer, M. De Chiara, S. Delmas, F. Dutreux, T. Fournier, A. Friedrich, E. Kornobis, J. Li, Z. Miao, L. Tattini, J. Schacherer, G. Liti, G. Fischer, Telomere-to-telomere assemblies of 142 strains characterize the genome structural landscape in *Saccharomyces cerevisiae*. *Nat. Genet.* **55**, 1390–1399 (2023).

32. J. Li, E. Coïc, K. Lee, C.-S. Lee, J.-A. Kim, Q. Wu, J. E. Haber, Regulation of budding yeast mating-type switching donor preference by the FHA domain of Fkh1. *PLoS Genet.* **8**, e1002630 (2012).

33. E. Sharon, S.-A. A. Chen, N. M. Khosla, J. D. Smith, J. K. Pritchard, H. B. Fraser, Functional genetic variants revealed by massively parallel precise genome editing. *Cell* **175**, 544–557.e16 (2018).

34. X. Guo, A. Chavez, A. Tung, Y. Chan, C. Kaas, Y. Yin, R. Cecchi, S. L. Garnier, E. D. Kelsic, M. Schubert, J. E. DiCarlo, J. J. Collins, G. M. Church, High-throughput creation and functional profiling of DNA sequence variant libraries using CRISPR–Cas9 in yeast. *Nat. Biotechnol.* **36**, 540–546 (2018).

35. K. R. Roy, J. D. Smith, S. Li, S. C. Vonesch, M. Nguyen, W. T. Burnett, K. M. Orsley, C.-S. Lee, J. E. Haber, R. P. St.Onge, L. M. Steinmetz, Dissecting quantitative trait nucleotides by saturation genome editing. bioRxiv 577784 [Preprint] (2024). https://doi.org/10.1101/2024.02.02.577784.

36. S. M. Richardson, L. A. Mitchell, G. Stracquadanio, K. Yang, J. S. Dymond, J. E. DiCarlo, D. Lee, C. L. V. Huang, S. Chandrasegaran, Y. Cai, J. D. Boeke, J. S. Bader, Design of a synthetic yeast genome. *Science* **355**, 1040–1044 (2017).

37. Y. Zhao, C. Coelho, A. L. Hughes, L. Lazar-Stefanita, S. Yang, A. N. Brooks, R. S. K. Walker, W. Zhang, S. Lauer, C. Hernandez, J. Cai, L. A. Mitchell, N. Agmon, Y. Shen, J. Sall, V. Fanfani, A. Jalan, J. Rivera, F.-X. Liang, J. S. Bader, G. Stracquadanio, L. M. Steinmetz, Y. Cai, J. D. Boeke, Debugging and consolidating multiple synthetic chromosomes reveals combinatorial genetic interactions. *Cell* **186**, 5220–5236.e16 (2023).

38. J. Dymond, J. Boeke, The *Saccharomyces cerevisiae* SCRaMbLE system and genome minimization. *Bioeng. Bugs* **3**, 168–171 (2012).

39. J. L. Doman, A. Raguram, G. A. Newby, D. R. Liu, Evaluation and minimization of Cas9-independent off-target DNA editing by cytosine base editors. *Nat. Biotechnol.* **38**, 620–628 (2020).

40. Z. Chen, J. K. Tyler, The chromatin landscape channels DNA double-strand breaks to distinct repair pathways. *Front. Cell Dev. Biol.* **10**, 909696 (2022).

41. R. Schep, E. K. Brinkman, C. Leemans, X. Vergara, R. H. van der Weide, B. Morris, T. van Schaik, S. G. Manzo, D. Peric-Hupkes, J. van den Berg, R. L. Beijersbergen, R. H. Medema, B. van Steensel, Impact of chromatin context on Cas9-induced DNA double-strand break repair pathway balance. *Mol. Cell* **81**, 2216–2230.e10 (2021).

42. W.-H. Chung, Z. Zhu, A. Papusha, A. Malkova, G. Ira, Defective resection at DNA double-strand breaks leads to de novo telomere formation and enhances gene targeting. *PLoS Genet.* **6**, e1000948 (2010).

43. M. Ricchetti, B. Dujon, C. Fairhead, Distance from the chromosome end determines the efficiency of double strand break repair in subtelomeres of haploid yeast. *J. Mol. Biol.* **328**, 847–862 (2003).

44. A. W. I. Lo, C. N. Sprung, B. Fouladi, M. Pedram, L. Sabatier, M. Ricoul, G. E. Reynolds, J. P. Murnane, Chromosome instability as a result of double-strand breaks near telomeres in mouse embryonic stem cells. *Mol. Cell. Biol.* **22**, 4836–4850 (2002).

45. O. Zschenker, A. Kulkarni, D. Miller, G. E. Reynolds, M. Granger-Locatelli, G. Pottier, L. Sabatier, J. P. Murnane, Increased sensitivity of subtelomeric regions to DNA double-strand breaks in a human cancer cell line. *DNA Repair* **8**, 886–900 (2009).

46. R. Lu, H. A. Pickett, Telomeric replication stress: The beginning and the end for alternative lengthening of telomeres cancers. *Open Biol.* **12**, 220011 (2022).

47. J. Peter, M. De Chiara, A. Friedrich, J.-X. Yue, D. Pflieger, A. Bergström, A. Sigwalt, B. Barre, K. Freel, A. Llored, C. Cruaud, K. Labadie, J.-M. Aury, B. Istace, K. Lebrigand, P. Barbry, S. Engelen, A. Lemainque, P. Wincker, G. Liti, J. Schacherer, Genome evolution across 1,011 *Saccharomyces cerevisiae* isolates. *Nature* **556**, 339–344 (2018).

48. A. M. Sriramachandran, G. Petrosino, M. Méndez-Lago, A. J. Schäfer, L. S. Batista-Nascimento, N. Zilio, H. D. Ulrich, Genome-wide nucleotide-resolution mapping of DNA replication patterns, single-strand breaks, and lesions by GLOE-Seq. *Mol. Cell* **78**, 975–985.e7 (2020).

49. L. Davis, N. Maizels, Homology-directed repair of DNA nicks via pathways distinct from canonical double-strand break repair. *Proc. Natl. Acad. Sci. U.S.A.* **111**, E924–E932 (2014).

50. C. Schwartz, J.-F. Cheng, R. Evans, C. A. Schwartz, J. M. Wagner, S. Anglin, A. Beitz, W. Pan, S. Lonardi, M. Blenner, H. S. Alper, Y. Yoshikuni, I. Wheeldon, Validating genome-wide CRISPR-Cas9 function improves screening in the oleaginous yeast *Yarrowia lipolytica*. *Metab. Eng.* **55**, 102–110 (2019).

51. E. Chen, E. Lin-Shiao, M. Trinidad, M. Saffari Doost, D. Colognori, J. A. Doudna, Decorating chromatin for enhanced genome editing using CRISPR-Cas9. *Proc. Natl. Acad. Sci. U.S.A.* **119**, e2204259119 (2022).

52. J. Ferreira da Silva, G. P. Oliveira, E. A. Arasa-Verge, C. Kagiou, A. Moretton, G. Timelthaler, J. Jiricny, J. I. Loizou, Prime editing efficiency and fidelity are enhanced in the absence of mismatch repair. *Nat. Commun.* **13**, 760 (2022).

53. M. Arbab, M. W. Shen, B. Mok, C. Wilson, Ż. Matuszek, C. A. Cassa, D. R. Liu, Determinants of base editing outcomes from target library analysis and machine learning. *Cell* **182**, 463–480.e30 (2020).

54. J. Koeppel, J. Weller, E. M. Peets, A. Pallaseni, I. Kuzmin, U. Raudvere, H. Peterson, F. G. Liberante, L. Parts, Prediction of prime editing insertion efficiencies using sequence features and DNA repair determinants. *Nat. Biotechnol.* **41**, 1446–1456 (2023).

55. F. Allen, L. Crepaldi, C. Alsinet, A. J. Strong, V. Kleshchevnikov, P. De Angeli, P. Páleníková, A. Khodak, V. Kiselev, M. Kosicki, A. R. Bassett, H. Harding, Y. Galanty, F. Muñoz-Martínez, E. Metzakopian, S. P. Jackson, L. Parts, Predicting the mutations generated by repair of Cas9-induced double-strand breaks. *Nat. Biotechnol.* **37**, 64–72 (2019).

56. J. Yin, M. Liu, Y. Liu, J. Wu, T. Gan, W. Zhang, Y. Li, Y. Zhou, J. Hu, Optimizing genome editing strategy by primer-extension-mediated sequencing. *Cell Discov.* **5**, 18 (2019).

57. F. P. Suchy, D. Karigane, Y. Nakauchi, M. Higuchi, J. Zhang, K. Pekrun, I. Hsu, A. C. Fan, T. Nishimura, C. T. Charlesworth, J. Bhadury, T. Nishimura, A. C. Wilkinson, M. A. Kay, R.

Majeti, H. Nakauchi, Genome engineering with Cas9 and AAV repair templates generates frequent concatemeric insertions of viral vectors. *Nat. Biotechnol.* **43**, 204–213 (2025).

58. S. Riesenberg, P. Kanis, D. Macak, D. Wollny, D. Düsterhöft, J. Kowalewski, N. Helmbrecht, T. Maricic, S. Pääbo, Efficient high-precision homology-directed repair-dependent genome editing by HDRobust. *Nat. Methods* **20**, 1388–1399 (2023).

59. F. Farzadfard, S. D. Perli, T. K. Lu, Tunable and multifunctional eukaryotic transcription factors based on CRISPR/Cas. *ACS Synth. Biol.* **2**, 604–613 (2013).

60. B. Bushnell, J. Rood, E. Singer, BBMerge—Accurate paired shotgun read merging via overlap. *PLOS ONE* **12**, e0185056 (2017).

61. C. Camacho, G. Coulouris, V. Avagyan, N. Ma, J. Papadopoulos, K. Bealer, T. L. Madden, BLAST+: Architecture and applications. *BMC Bioinformatics* **10**, 421 (2009).

62. M. Martin, Cutadapt removes adapter sequences from high-throughput sequencing reads. *EMBnet.journal* **17**, 10–12 (2011).

63. H. Li, R. Durbin, Fast and accurate short read alignment with Burrows-Wheeler transform. *Bioinforma. Oxf. Engl.* **25**, 1754–1760 (2009).

64. A. McKenna, M. Hanna, E. Banks, A. Sivachenko, K. Cibulskis, A. Kernytsky, K. Garimella, D. Altshuler, S. Gabriel, M. Daly, M. A. DePristo, The genome analysis toolkit: A MapReduce framework for analyzing next-generation DNA sequencing data. *Genome Res.* **20**, 1297–1303 (2010).

65. J. A. Wala, P. Bandopadhayay, N. F. Greenwald, R. O'Rourke, T. Sharpe, C. Stewart, S. Schumacher, Y. Li, J. Weischenfeldt, X. Yao, C. Nusbaum, P. Campbell, G. Getz, M. Meyerson, C.-Z. Zhang, M. Imielinski, R. Beroukhim, SvABA: Genome-wide detection of structural variants and indels by local assembly. *Genome Res.* **28**, 581–591 (2018).

66. M. Mahmoud, N. Gobet, D. I. Cruz-Dávalos, N. Mounier, C. Dessimoz, F. J. Sedlazeck, Structural variant calling: The long and the short of it. *Genome Biol.* **20**, 246 (2019).

67. J. T. Robinson, H. Thorvaldsdóttir, W. Winckler, M. Guttman, E. S. Lander, G. Getz, J. P. Mesirov, Integrative genomics viewer. *Nat. Biotechnol.* **29**, 24–26 (2011).

68. M. I. Love, W. Huber, S. Anders, Moderated estimation of fold change and dispersion for RNA-seq data with DESeq2. *Genome Biol.* **15**, 550 (2014).

69. N. V. Chawla, K. W. Bowyer, L. O. Hall, W. P. Kegelmeyer, SMOTE: Synthetic minority over-sampling technique. *J. Artif. Intell. Res.* **16**, 321–357 (2002).

70. H. He, Y. Bai, E. A. Garcia, S. Li, "ADASYN: Adaptive synthetic sampling approach for imbalanced learning," in *2008 IEEE International Joint Conference on Neural Networks (IEEE World Congress on Computational Intelligence)* (IEEE, 2008), pp. 1322–1328.

71. F. Mölder, K. P. Jablonski, B. Letcher, M. B. Hall, C. H. Tomkins-Tinch, V. Sochat, J. Forster, S. Lee, S. O. Twardziok, A. Kanitz, A. Wilm, M. Holtgrewe, S. Rahmann, S. Nahnsen, J. Köster, Sustainable data analysis with Snakemake. *F1000Res.* **10**, 33 (2021).

72. A. N. Schep, J. D. Buenrostro, S. K. Denny, K. Schwartz, G. Sherlock, W. J. Greenleaf, Structured nucleosome fingerprints enable high-resolution mapping of chromatin architecture within regulatory regions. *Genome Res.* **25**, 1757–1770 (2015).

73. A. Weiner, T.-H. S. Hsieh, A. Appleboim, H. V. Chen, A. Rahat, I. Amit, O. J. Rando, N. Friedman, High-resolution chromatin dynamics during a yeast stress response. *Mol. Cell* **58**, 371–386 (2015).

74. S. Venkatesh, H. Li, M. M. Gogol, J. L. Workman, Selective suppression of antisense transcription by Set2-mediated H3K36 methylation. *Nat. Commun.* **7**, 13610 (2016).
